# Ecological impacts of the LED-streetlight retrofit on insectivorous bats in Singapore

**DOI:** 10.1371/journal.pone.0247900

**Published:** 2021-05-26

**Authors:** Kenneth Ee Meng Lee, W. H. Deon Lum, Joanna L. Coleman

**Affiliations:** Department of Biological Sciences, National University of Singapore, Singapore, Republic of Singapore; Universidade Estadual de Santa Cruz - UESC, BRAZIL

## Abstract

Cities around the world are transitioning to more efficient lighting schemes, especially retrofitting traditional, high-pressure sodium (HPS) streetlights with light-emitting diode (LED) lights. Although these initiatives aim to address the problems of urban sustainability and save money, the ecological impacts of these retrofits remain poorly understood, especially in brightly lit cities and in the tropics, where urbanisation is most rapid. We performed an experimental study of the retrofit in Singapore–focusing on insectivorous bats, whose activity we monitored acoustically along paired control (HPS-lit) and treatment (LED-lit) streets. We recorded seven species along these streets, but only obtained enough recordings to measure the effect of light type for three of them–all of which can reasonably be described as urban adapters. The strongest predictor of bat activity (an index of habitat use) was rainfall–it has a positive effect. Light type did not influence bat activity or species composition of the bat assemblage along these streets, though it did interact with the effects of rainfall and traffic noise for one bat species. Ultimately, the retrofit may be ecologically meaningless to urban-adapted, tropical insectivores that already experience high levels of light pollution as they do in Singapore. However, while our findings may appear reassuring to those concerned with such retrofits in other tropical and/or brightly-lit cities, they also highlight the contextual nature of ecological impacts. We point out that they should not be prematurely generalised to other locales and systems. In particular, they do not imply no impact on species that are less urban-adapted, and there is a clear need for further studies, for example, on responses of other foraging guilds and of bats (and insects) throughout the tropics.

## Introduction

Urbanisation and artificial light at night (ALAN) are inexorably linked–hence the widespread use of remotely-sensed nighttime light as a metric of urbanisation [1]. Accordingly, given current and projected urbanisation, i.e., 1.6 times as many people living in cities by 2050 compared to 2018 [2], the pervasiveness of ALAN can reasonably be expected to increase. Already, ALAN affects ever larger proportions of Earth’s surface [roughly 2% per year; 3] and of humans [more than 80% of all people exposed to light pollution; 4].

Lighting up the night has many consequences, including biological and ecological ones, which are well-documented (albeit with some gaps). Certain impacts, such as the fatal attraction of moths to lamps or the disorientation of sea-turtle hatchlings on beaches fringed by lit buildings, are more well-known than others [5]. But impacts are documented in a vast literature and diversity of organisms ranging from fungi [e.g., 6] to plants [7] to marine invertebrates [e.g., 8] to large mammals [9]. These population- or community-level impacts should ultimately alter ecosystem structure and function [10].

Impacts on prey-predator relationships might seem especially observable in certain study systems, one being insectivorous bats, which, as the main predators of nocturnal insects, are key biocontrol agents [11]. Many nocturnal insects orient toward ALAN for reasons that remain somewhat unclear but may involve interference with their use of stars to navigate [10]. Decades of research have shown that ALAN may thus create advantageous conditions for some bats that actively hunting swarms of insects at lights [e.g., 12, 13]. However, it has apparently detrimental impacts on other species [e.g., 14, 15]. These differential responses of bats seem at least partly attributable to flight and foraging styles. Most so-called light-opportunistic species [e.g., by 16] are faster-flying aerial hawkers and adapted to foraging in open areas, whereas light-averse bats tend to be slower-flying and adapted for foraging in more cluttered microhabitats. Light-avoidance behaviour in open areas by slower-flying bats should be selected for given that light makes bats more visible to visual predators [e.g., 17], although more evidence is needed to support this hypothesis [see also 18].

ALAN affects insectivorous bats at least by influencing the distribution of their prey and/or their perceived predation risk. However, most studies have examined the impacts of single, traditional lighting technologies, especially of the high-pressure sodium (HPS) vapour lamps that have dominated most installed outdoor lighting capacity [19, 20]. Consequently, there is less understanding of the role of light type, which should be ecologically meaningful given that insects and vertebrates vary in their sensitivities to different wavelengths of light [21]. Generating such knowledge is timely amid a widespread shift toward light-emitting diode (LED) technology, which offers many advantages over traditional light types. These include energy and cost savings, reduced lifetime CO_2_ emissions and the ability to be controlled and integrated into smart lighting schemes [20]. As for spectral properties, whereas HPS lamps emit a yellowish-orange light that peaks most strongly at 819 nm, LEDs emit a broader spectrum of wavelengths between 400 and 700 nm [22]. The cost-effectiveness and potential contribution to climate-change mitigation [e.g., 19] explain why many cities and other jurisdictions are undertaking LED-streetlight retrofits.

With these retrofits proceeding apace in the 2010s, there has been a flurry of research interest in how LEDs affect insectivorous bats and insects. Results are mixed. In the UK, LED lights inhibited slow- but not faster-flying bats relative to dark controls [23]. A retrofit from low-pressure sodium (LPS) to LEDs in the UK had no apparent effects on bats [24], although a re-analysis of the data [25] suggests more nuanced effects–negative impacts were apparent, but only at high light intensities. Studies of retrofits from mercury-vapour (MV) to LEDs have revealed: negative impacts on light-tolerant bats and positive impacts on light-averse species in six German cities [18] and negative impacts on clutter- and edge-adapted bats in Sydney, Australia [26]. As for effects of LEDs on insects, they attracted more of them than HPS lights did in New Zealand [27], whereas in Germany, they attracted fewer insects than MV lights did, especially under urban conditions [28]. Finally, LED lights may interfere with the evolved flight manoeuvres that allow moths to evade predation by bats [29].

The research described above underscores the likelihood that the impacts of ALAN in general (and LED retrofits in particular) are highly contextual. Yet, certain contexts remain largely unaddressed. One is the large, brightly lit city–except for two of six urban areas sampled by Lewanzik and Voigt [18]. This is potentially problematic because the impact of an individual light source is magnified in darker overall conditions [e.g., 12]. The other unaddressed context is the tropical city. It is more concerning because bats (like most organisms) are most speciose in the tropics [30] and tropical bats are the least well-studied bats when it comes to the impacts of anthropogenic habitat changes in general [31]. Most importantly, the tropics, especially in the Old World, are where urbanisation is happening fastest [2]. With large, brightly-lit cities in the tropics implementing LED retrofits, we consider these unaddressed contexts to represent significant research gaps.

We conducted an experimental study of the impacts of the retrofit on insectivorous bats in Singapore. This island city-state is the world’s most light polluted nation [4] and the only fully urbanised tropical nation [with 100% of residents in urban areas; 2]. Despite having experienced the most severe deforestation of any Southeast (SE) Asian nation [32], Singapore still has many species of insectivorous bats, and though some are restricted to the tiny remaining fragments of primary forest, several occur in more urbanised habitats [33].

At the time of our study, the government was partway through an island-wide streetlight retrofit to replace all 95 000 existing HPS streetlamps with LEDs by 2022 [34]. We took advantage of the partially completed work to create an experimental study, comparing bats and their foraging activity between streets with the newer LED lamps (treatment) and streets with the old, HPS lamps (controls). Given the expectation that LED lights are more attractive than sodium-vapour lights to insects [as in 27], we hypothesised that the retrofit benefits bats that use streets as foraging habitats. Accordingly, we predicted that bat activity and foraging are higher at treatment streets than at controls. We also predicted that species composition of the assemblage is more dominated by bats presumed to be light-opportunistic (aerial hawkers) at treatment streets.

## Methods

### Study area

Singapore (1.3521° N, 103.8198° E) is a sovereign island nation, south of Peninsular Malaysia. In 2017, it had a land area of 721.5 km^2^ [35] and a population of 5.6 million people [36]. As mentioned, it is the only tropical nation with its entire human population urbanised [2] and the world’s most brightly-lit nation [4].

Singapore’s bat fauna includes five species of Old World fruit bats (family Pteropodidae) and 15 insectivorous bats, most of which are uncommon or locally endangered [37].

### Experimental design

We designed our study following two British studies [24, 38]. To isolate the effects of the retrofit from those of extraneous temporal and environmental variables [see also 39], we aimed control and impact conditions must be as similar as possible.

**Fig 1:**
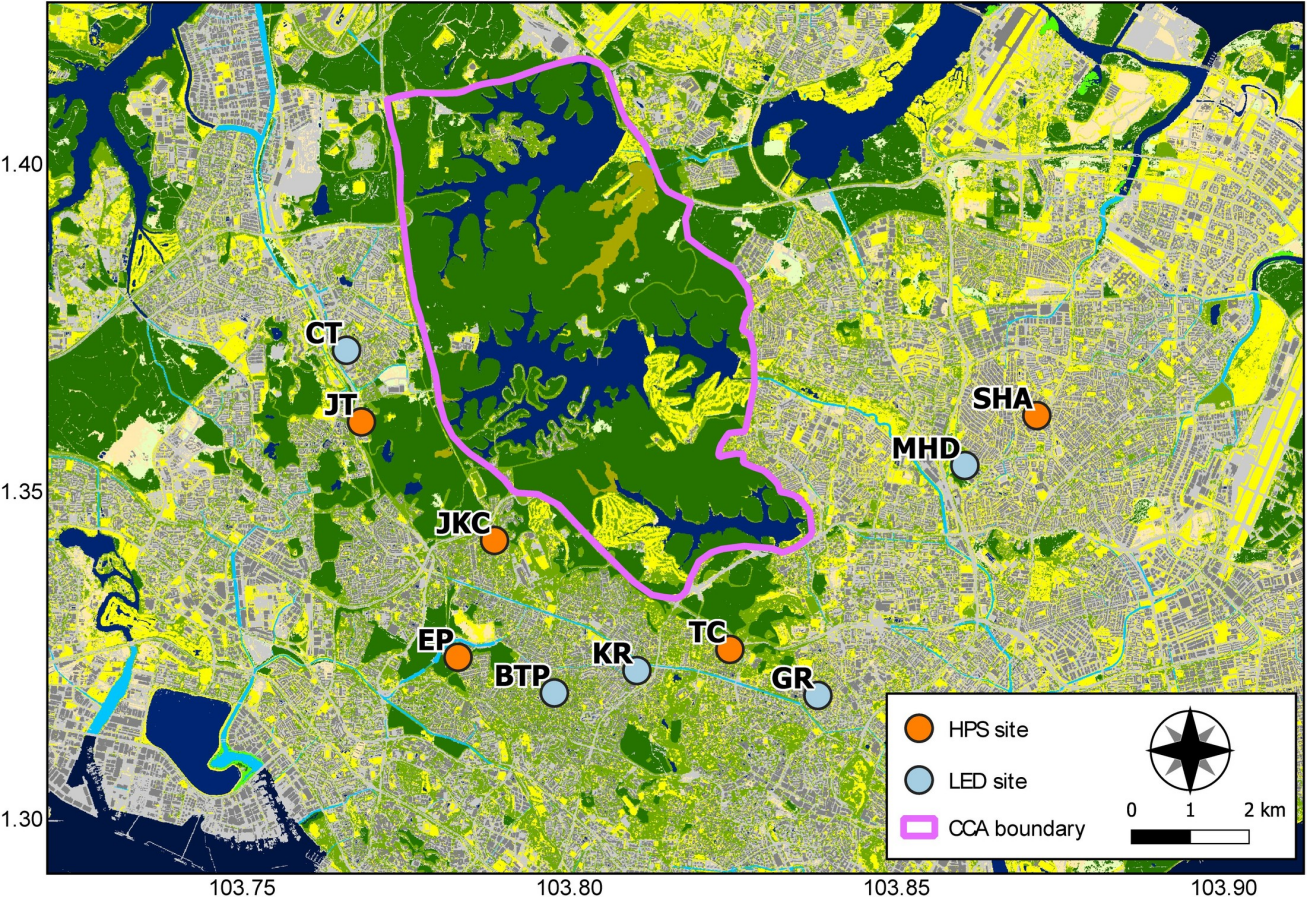
Location of HPS (control) and LED (treatment) streets. Base map taken from Gaw et al. [40]. Polygon delineates the boundary of the Central Catchment Area.

After consulting a map of the retrofit progress provided by the Land Transport Authority of Singapore (LTA), we chose 10 sites: five control replicates and five treatment replicates (Fig 1). Throughout the study, each control street was lit by the old HPS lights; each treatment street had already had LED lights installed. To ensure independence (minimise the likelihood of recording the same individuals at control and treatment streets), all streets were at least 1.2 km (M = 1.8 km, SD = 0.7 km) apart.

To reduce any potential influence of the urbanisation gradient, we only selected sites in residential areas dominated by landed properties, which are privately-owned, detached or semi-detached (sharing a common wall) dwellings that are two to three storeys high and on fenced lots. Also, using qGIS 3.14.15 [41] and the base map from Gaw et al [40], we established that all sites were relatively near (M = 2.1 km, SD = 1.0 km) the Central Catchment Area, which is roughly in the centre of the island and contains most of Singapore’s primary forest–distance to primary forest being a common, local urbanisation-gradient metric [e.g., 42] and a variable that is ecologically meaningful to bats [33]. Finally, they were all within 0.8 km (M = 0.4 km, SD = 0.2 km) from the nearest permanent freshwater body (river, canal, reservoir), although smaller anthropogenic water bodies, such as fountains and pools, which we have personally observed bats using, may have been closer.

Using lighting specifications provided by LTA, we matched control and treatment streets for light height, power output, colour temperature and illuminance. All lights were 6 m tall. We obtained colour temperature values from LTA–they were 2100 K (HPS) and 4000 K (LED). We measured illuminance using a lux meter–values were 30 lux (HPS) and 50 lux (LED).

### Acoustic monitoring

We conducted fieldwork from October 2017 through January 2018. Each night (sampling event), we monitored bats simultaneously at one treatment and one control street. We sampled each site five times on randomly selected nights, with roughly one month between sampling events at each site. This ensured a balanced study design with roughly equal sample sizes for the inter-monsoonal (October-November) and early Northeast Monsoon (December-January) periods [43], but we never sampled on nights with rain.

We recorded bats using brand new acoustic detectors (Anabat Express; Titley Scientific), which we mounted on randomly selected lampposts, by securing them at a height of 1.5 m above the ground, with the microphone angled upward at 25° toward the lamp. We set the detectors to record for two hours continuously, starting at sunset, with these settings: high sensitivity, division ratio of 8; maximum recording time 15 seconds. The detectors store files as zero crossing analysis (ZCA) files.

Various abiotic factors can affect activity of tropical bats. These include moon phase and minimum nightly temperature [44]. However, we did not expect these variables to be ecologically meaningful in our study. Singapore’s bright ambient light [4] should reduce, if not, negate any influence of moonlight, especially along lit streets. And there is almost no temporal variation in minimum temperature [43]. However, we did expect potential confounding effects of traffic noise [e.g., 45] and rain, which could conceivably affect the availability of insect prey [e.g., 44]. Therefore, we recorded traffic noise (dB) using an app (Decibel X) on an iPhone 6 and obtained government data on total monthly rainfall [46].

### Bat call identification

We interpreted 100 hours of recordings using AnalookW (v. 4.2n, Titley Scientific), which allows echolocation calls to be visualised on a spectrogram and listened to (by converting ZCA files to WAV files). Thus, we inspected each file by sight and ear simultaneously–a method that reduces the likelihood of missing calls that are audible but not visible, which may happen when using frequency-division [47]. We used search phase calls [following 48] to identify passes to species. Because there was no reference call library for bats in Singapore, or even SE Asia, we based identifications on published call data from a small-scale, local study [33] and expert verification (BPY-H Lee and third author, both pers. comm.). Compared to the ZCA detectors we used, full-spectrum detectors, as employed by Pottie et al. [33] generally record more calls and offer greater resolution [49]. For species-identification purposes, we expect those differences to be minimal in our context, but we identified calls based on measured parameters, such as minimum frequency, slope and duration [see also 47]. In some cases, we could not assign species epithets to calls, but could assert (based on unique call parameters) that they belonged to species other than the ones we could confidently identify. We named such passes unknown.

Acoustic monitoring does not allow researchers to directly estimate abundance, but rather to quantify site use (as bat activity) and foraging activity–two useful indicators of the impacts of environmental change [50]. As in other studies [e.g., 24, 50], we quantified bat activity as the number of passes per night, defining a pass as a sequence of at least two search-phase calls no more than one second apart and with individual passes separated by at least one second of silence. We quantified foraging activity as the feeding buzz ratio (total feeding buzzes divided by total passes). Feeding buzzes are stereotypical sequences of calls with decreasing inter-call intervals that bats emit as they zero in on a potential prey item. We quantified bat activity for the entire bat assemblage (total) and each species separately.

### Statistical analysis

We estimated three response variables: (1) bat activity (number of passes), (2) feeding buzzes and (3) species composition. We performed all statistical analyses in R 3.6.2 [51] at a significance level of α = 0.05 (data in S1 Appendix, R code in S2 Appendix).

#### Bat activity

To assess whether bat activity differed between the control and treatment, we used a generalised linear mixed effects model (GLMM), with total passes (i.e., from all species) as the response variable, light-type, monthly rainfall and traffic noise as fixed effects and site as a random effect. We normalised monthly rainfall and traffic noise using the ‘scale’ function in the base ‘stats’ package to help with model convergence.

We initially used a Poisson distribution with glmer() in the lme4 package [52], but the model was overdispersed, so we opted to use the more flexible negative binomial distribution with glmer.nb() from the same package. We started with a fully-crossed model and removed non-significant interaction terms sequentially. We used the vif() from the car package [53] to establish that there was no multicollinearity among remaining terms (defined as variance inflation factors > 5). We evaluated model fit visually with diagnostic plots produced with simulateResiduals() from the DHARMa package [54].

To assess possible effects of light-type on individual species, we performed separate analyses for the three most common species: *Saccolaimus saccolaimus*, *Myotis muricola*, and *Scotophilus kuhlii*. Because we recorded at least 1 000 passes of each of these bats across all replicates, they were more amenable to statistical analysis than rare species. We used the same model structure and tests as above, but with species-specific passes as the dependent variable. For *M*. *muricola*, the model failed to converge, even after removing terms (probably because of the many nights when we did not record it), so we dropped the species from this analysis.

#### Feeding buzzes

Although we set out to investigate the effect of the retrofit on foraging activity, we recorded so few feeding buzzes that a statistical comparison between the control and treatment would have been ecologically meaningless and impractical.

#### Species composition

We compared species composition across different light treatments using non-metric multidimensional scaling (NMDS) with metaMDS() from the vegan package [55]. We assessed the fit qualitatively for different dimensions using scree plots. We opted to use k = 2 dimensions, which had a reasonable stress of 0.159 and would facilitate interpretation of results. Finally, we plotted 95% CI ellipses to check for overlaps between factor levels.

## Results

Over 25 sampling nights, we recorded a total of 8 409 passes and 215 feeding buzzes from seven species: *Myotis muricola*, *Saccolaimus saccolaimus*, *Scotophilus kuhlii*, *Taphozous melanopogon*, *Rhinolophus lepidus* and two unknown bats.

### Bat activity

**Fig 2:**
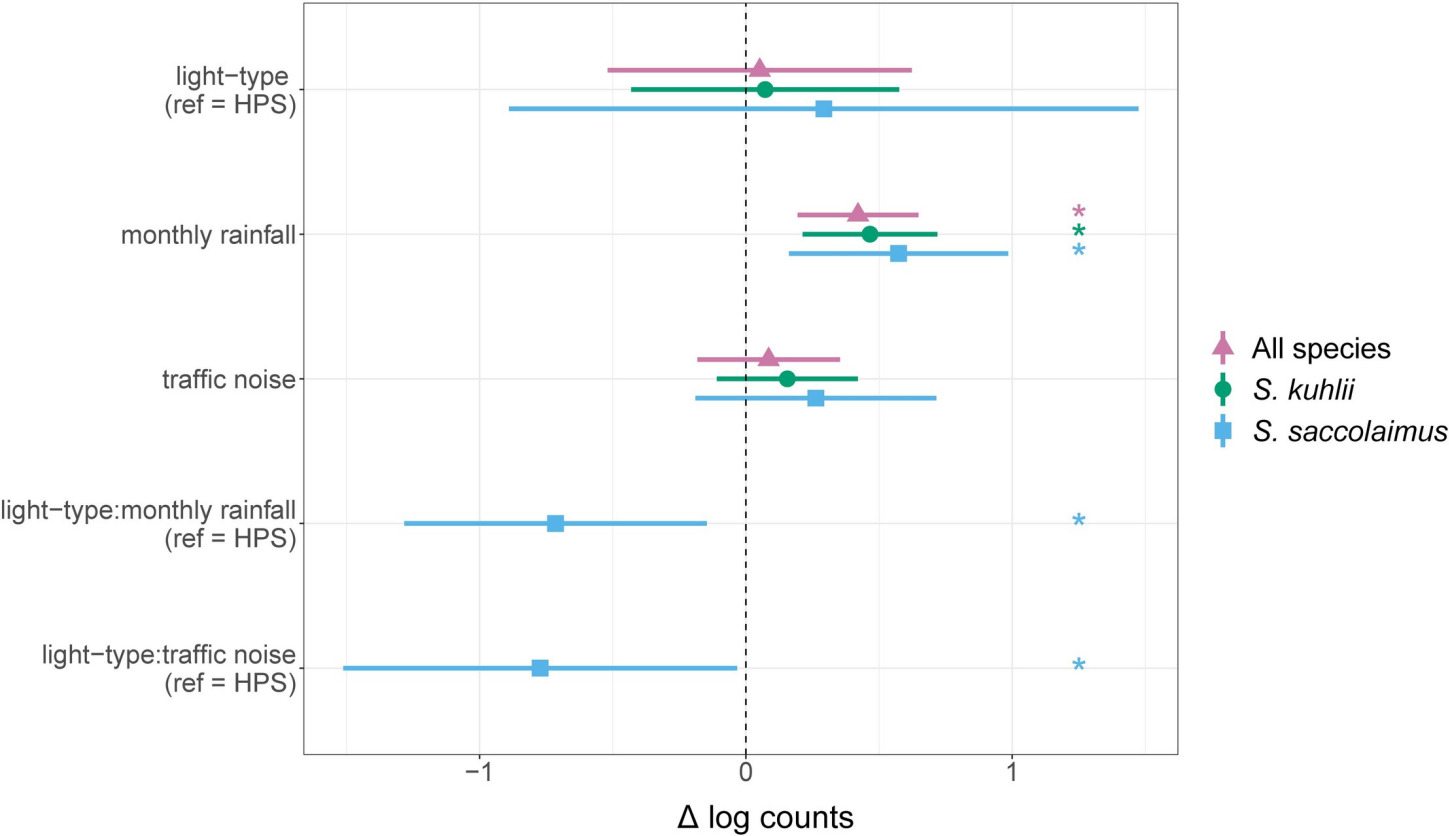
Effects of variables retained in the final GLMM model on log counts of bat passes for: All species, *Scotophilus kuhlii*, *Saccolaimus saccolaimus*. Dots are point estimates, with whiskers showing the 95% confidence interval. Asterisks beside dot-whiskers indicate significant effects. The reference level for light type was HPS (i.e., the change indicates the change when moving from an HPS to a LED street). Monthly rainfall and traffic noise were normalised to help with model convergence.

The vast majority (> 95%) of passes were from three species: *S*. *kuhlii* (n = 5 538), *M*. *muricola* (n = 1 395), and *S*. *saccolaimus* (n = 1 088). In contrast to *S*. *kuhlii* and *S*. *saccolaimus*, which we detected on almost every sampling event and whose activities were fairly evenly distributed among events, that of *M*. *muricola* was distributed unevenly, with 664 passes recorded during two sampling events (site BTP night 3, site TC night 3). Total numbers of passes were similar between treatment and control conditions: 4 322 on all LED-lit streets and 4 087 on all HPS-lit streets. In our final GLMM model, light-type (incident-rate-ratio (IRR) = 1.05, p = 0.858) and traffic noise (IRR = 1.09, p = 0.532) did not significantly affect overall bat activity (Fig 2; S3 Appendix). Only monthly rainfall was statistically significant (IRR = 1.52, p < 0.001).

Our subsequent analysis of species-specific effects, i.e., zeroing in on two of the three most common bats revealed that light type and traffic noise did not affect the activity of either of them (Fig 2) while monthly rainfall was a significant predictor for both (*S*. *kuhlii*: IRR = 1.59, p < 0.001; *S*. *saccolaimus*: IRR = 1.77, p = 0.006; S3 Appendix). For *S*. *saccolaimus*, the final model retained two significant interactions: light-type X monthly rainfall and light-type X traffic noise. In both cases, the interaction was cross-over in nature. In other words, the effect of monthly rainfall or traffic noise on bat activity was positive on HPS-lit streets and negative at LED-lit streets (S4 Appendix).

### Foraging activity

Foraging activity was low–only 2.6% of all passes contained feeding buzzes. We also only detected feeding buzzes from the same three common species mentioned above. Most were from *S*. *kuhlii* (n = 110) and *M*. *muricola* (n = 100)–only five were from *S*. *saccolaimus*. Finally, we detected 50% more feeding buzzes (n = 129) on treatment (LED) than on control (HPS) streets (n = 86).

### Species composition

**Fig 3:**
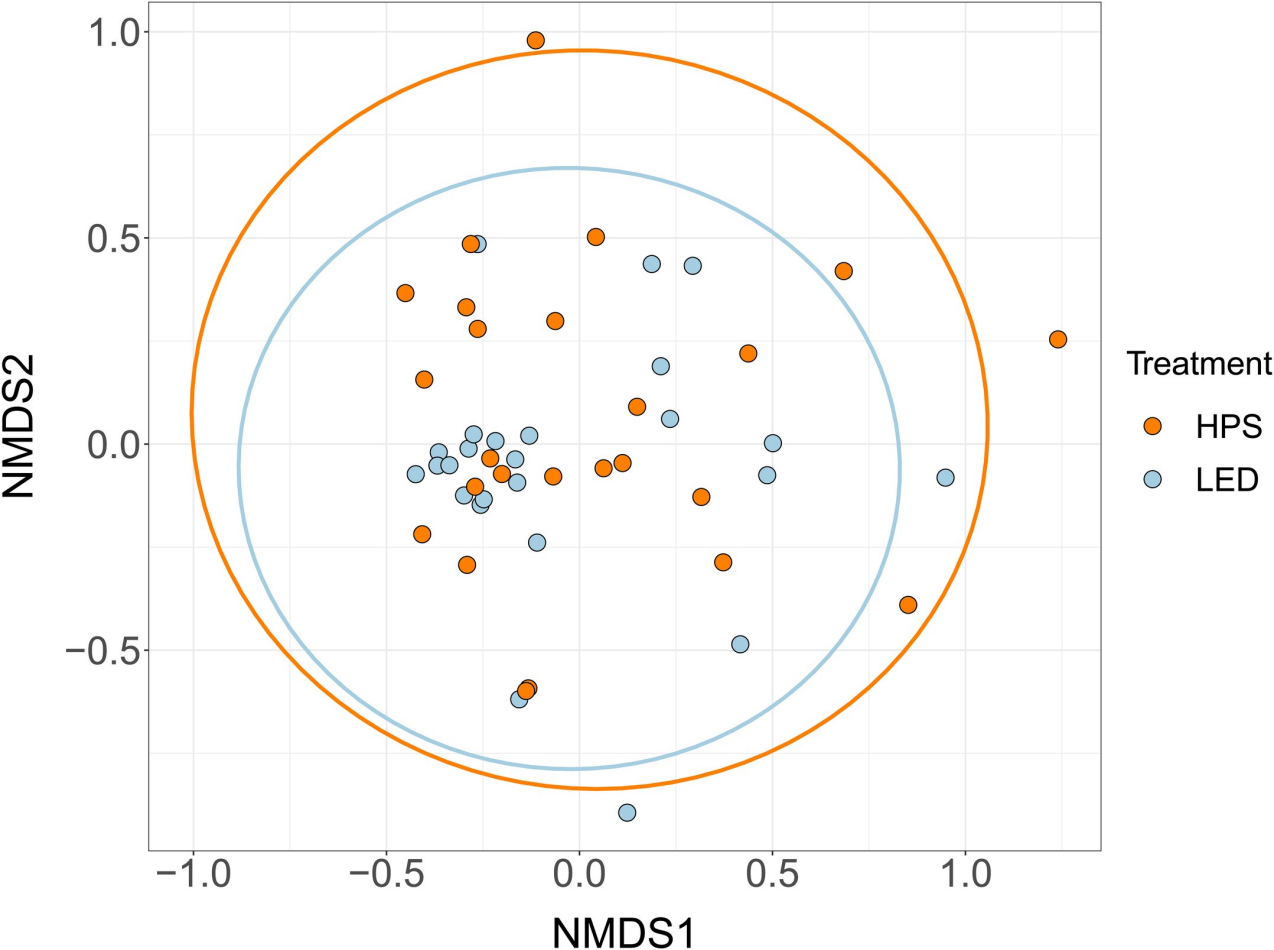
NMDS plot generated from Bray-Curtis dissimilarities. The nearly complete overlap between 95% confidence ellipses (solid lines) for both light treatments suggests similar species compositions.

We detected six of all seven species along both treatment and control streets. The seventh (*R*. *lepidus*) was very rare–we only detected it twice along one control street. Our NMDS plots (Fig 3) with 95% confidence ellipses suggested no difference in species composition between HPS and LED streets.

## Discussion

To investigate the impacts of an LED streetlight retrofit on insectivorous bats in Singapore, we compared bat activity between treatment streets where the retrofit was complete and control streets that still retained the old, HPS lights, and found no significant differences. Thus, the retrofit does not seem to affect insectivorous bats (or at least not the species using these streets), and we find no evidence to support our hypothesis that it benefits them (which was based on our expectation that LEDs attract more insect prey). Although this finding seems similar to those of the only Before-After-Control-Impact-Paired (BACIP) study of the HPS-to-LED retrofit on bats [24], which recorded no impacts on open-space foragers, a robust re-analysis of its data [25] revealed more nuanced effects–namely, they strongly depended on illuminance, and the retrofit (at least at lower light intensity) did produce higher activity of so-called light tolerant species.

We similarly found no support for our prediction that species composition along LED-lit streets (due to the above-mentioned, prey-related expectation) is more skewed toward bats whose flight styles and foraging guilds are more adapted to hunting in open habitats [also see 13]. In fact, the retrofit had no effect on assemblage composition. Still, the most common bats along both types of streets (*S*. *kuhlii* and *S*. *saccolaimus*) are, indeed, fast-flying, aerial hawkers [33], while the rarest one, *R*. *lepidus*, is specialised for cluttered, forest environments [56]. We were, however, surprised by the high number of passes for *M*. *muricola* given that it is adapted for slow, manoeuvrable flight in cluttered habitats [33]. As mentioned, nearly 48% of *M*. *muricola* activity occurred on two nights and this bat accounted for nearly 46% of all foraging activity. We suspect that we actually recorded a few foraging individuals on those nights–this could have positively skewed the proportional representation of this bat in our study. It is also worth noting that *M*. *muricola* often roosts inside furled banana leaves [33], and there were banana plants growing on the side of many of our streets.

Ultimately, we attribute the lack of support for our overall hypothesis and its predictions to the very low foraging activity we observed. This was surprising considering the well-documented phenomenon of bats hunting insects at street lamps, including in the tropics [57]. In any event, the bats we recorded were evidently using streets mainly to commute–not hunt. Therefore, even if there is a difference between HPS and LED lights in their attractiveness to insects (a question for future research), it appears ecologically meaningless to these bats. As to why bats were not using these streets to forage, we propose that with all the brightly lit roads in Singapore, bats are spoiled for choice and there are more profitable lights elsewhere. Indeed, a prior study (S5 Appendix) that recorded much higher feeding buzz ratios than ours tested the effect of artificial light on bats at a pond in a large park.

We also detected no impact of traffic noise on bat activity. This is unexpected considering that controlled studies [i.e., in vitro or field-playback; 45, 58–61] find that it is detrimental to diverse bats, specifically to foraging. In the field, bats could be attracted to roadside habitats if streetlights attract insect prey and bats can still detect and hunt them [as proposed by 62]. However, although such a phenomenon may be expected in largely dark (e.g., peri-urban) landscapes, with Singapore so brightly lit, it seems unlikely to explain the apparent insensitivity of bats to noise that we observed. Instead, we reiterate that the bats we recorded were mainly commuting. Moreover, traffic noise in all sites was high (ca. 60 dB) and we did not have quiet controls without traffic noise. Perhaps, for animals already used to commuting along Singapore’s roads, additional variation in traffic noise is simply not ecologically meaningful.

The best predictor of bat activity was monthly rainfall. We recorded more bats during the rainiest months, which correspond to the wet phase of the Northeast Monsoon [43]. Though this relationship has never been studied in Singapore, it does not seem unusual for the tropics. Most likely, rain increases the availability of insects in general and, in turn, the activity of bats. Indeed rainfall may be the most important abiotic determinant of insect population dynamics in the tropics [63], and positive correlations between rainfall and insect abundance have been observed in studies of bats in tropical Africa [64], Mexico [65], Malaysia [66] and in a concurrent local study (S6 Appendix). The seasonal change in bat activity might also reflect reproductive phenology. More specifically, lactation [the most energetically costly stage of reproduction; e.g., 66, 67] and/or weaning [e.g., 64] may be timed to match the period of peak prey availability.

What is harder to explain are our observed interactive effects of light type on the activity of *S*. *saccolaimus*, which increased with traffic noise and rainfall along HPS-lit streets and decreased with both variables along LED-lit streets. Moreover, we failed to observe these effects for *S*. *kuhlii*, even though both bats are high, fast-flying aerial insectivores that seem equally well-adapted to urban life [33]. Perhaps the answer lies in the link between weather and sound transmission. The main weather variable that varies seasonally in Singapore (besides rainfall) is wind–the rainiest months (when bat activity peaked) are also the windiest [43]. The relationship between weather and sound transmission is complex, but urban sound propagation may vary with wind [68], and weather may alter how bats echolocate and/or perceive sound [69]. The literature on sounds emitted by various streetlighting technologies is scant, but HPS and LEDs may differ in that respect [70]. We could not measure noise produced by streetlamps, but we wonder if it might interact with the sound of rain and/or traffic in a way that matters to bats.

Overall, we find no evidence that the LED streetlight retrofit in Singapore has ecological impacts on insectivorous bats that exceed those of the old HPS lights. Given that the retrofit contributes to sustainable urbanisation, this is potentially encouraging. However, we acknowledge certain caveats. First, we collected data over one four-month period, and although we timed it to evenly sample two intervals that differ in rainfall, bats may respond differently at other times of the year depending, for instance, on phenology. We therefore recommend that future studies be conducted over at least one year. Second, we focused solely on insectivorous bats because their use of vocal echolocation facilitates passive acoustic monitoring. However, Singapore has at least four species of Old-World fruit bats (family Pteropodidae) [37], two of which (*Cynopterus brachyotis* and *Eonycteris spelaea*) often roost, commute and forage along roads (pers. obs.). Two studies [71, 72] have shown detrimental impacts of ALAN on neotropical fruit bats (family Phyllostomidae), but there is no knowledge of how pteropodids respond or whether lighting technology matters. We believe this represents a key conservation and ecosystem-service research gap. For instance, in Singapore, *C*. *brachyotis*, may be a key seed disperser [42] but has undergone an extreme decline in genetic diversity [73] that may portend extirpation. And *E*. *spelaea* is a vital pollinator in various habitats in SE Asia [74–76]. Therefore, we strongly encourage studies using methods suited to fruit bats. Third, it should be noted that our use of ZCA (as opposed to full-spectrum) detectors may have limited our ability to detect certain species whose calls are typified by steep, FM sweeps [49]. However, based on occurrence data in the paper we used as a reference for bat identification [33], we did not expect to find these bats along roads. Full-spectrum detectors may also record more feeding buzzes [49]. However, in reviewing each file visually and by sound, we aimed to mitigate this issue. We therefore acknowledge that we may have missed some rare species and/or foraging activity, but expect that this minimally, if at all, affected our findings. Finally, we studied the impacts of a retrofit in progress, not the installation of new lights in a formerly dark area. The impacts of ALAN in general on bat activity may still persist in our study area and elsewhere and represent an ongoing research priority.

In conclusion, although some studies [18, 23, 24, 26] have tested the effects of LED lights on bats, none have done so in the tropics, despite their unparalleled bat diversity. Moreover, this issue has rarely been addressed in large, brightly lit cities. These are the two research gaps we addressed in Singapore, the world’s most light-polluted nation [4]. The fact that our findings differed from those reported elsewhere illustrates that the impacts of widespread streetlamp retrofits may be contextual, and that effects documented in the temperate zone or relatively dark areas poorly generalisable. We again reiterate the need for studies in large, brightly lit urban areas and in the tropics. We especially encourage researchers to carry out similar studies in SE Asia, which is urbanising very quickly [2] and has nearly one quarter of global bat diversity and whose bats have a very high rate of endangerment [77].

## Supporting information

S1 Appendix Study data used to test hypotheses (i.e., results of each sampling event). (XLSX)

S2 Appendix R code. (R)

S3 Appendix Regression analysis (GLMM output). (DOCX)

S4 Appendix Plots of interactions of light type with rainfall and traffic noise for *Saccolaimus saccolaimus* with 95% confidence intervals. (PDF)

S5 Appendix Unpublished thesis (The effect of an experimental light treatment on bat activity over an urban water body) by Michelle GAN Wan Jie. (PDF)

S6 Appendix Unpublished thesis (The switch from HPS to LED streetlamps–impacts on insects) by YAO Xinyi. (PDF)
