# Supplementary material for: Ecological impacts of the LED-streetlight retrofit on insectivorous bats in Singapore
Source: PLoS One. 2021 May 26;16(5):e0247900. doi: 10.1371/journal.pone.0247900 (PMC8153503; doi:10.1371/journal.pone.0247900)
Supplement: S3 Appendix — (DOCX) [file pone.0247900.s003.docx]

**GLMM output for total passes (i.e. all species included)**

Generalized linear mixed model fit by maximum likelihood (Laplace Approximation) ['glmerMod']

Family: Negative Binomial(1.6856) ( log )

Formula: total_passes ~ light_type + monthly_rainfall + traffic_db + (1 | site)

Data: bat_data

AIC BIC logLik deviance df.resid

609.5 620.9 -298.7 597.5 44

Scaled residuals:

Min 1Q Median 3Q Max

-1.1382 -0.7261 -0.2379 0.4420 3.3458

Random effects:

Groups Name Variance Std.Dev.

site (Intercept) 0.1047 0.3236

Number of obs: 50, groups: site, 5

Fixed effects:

Estimate Std. Error z value Pr(>|z|)

(Intercept) 4.94079 0.21389 23.100 < 2e-16 ***

light_typeLED 0.05786 0.22568 0.256 0.79767

monthly_rainfall 0.43213 0.11233 3.847 0.00012 ***

traffic_db 0.10955 0.14787 0.741 0.45877

---

Signif. codes: 0 ‘***’ 0.001 ‘**’ 0.01 ‘*’ 0.05 ‘.’ 0.1 ‘ ’ 1

Correlation of Fixed Effects:

(Intr) lg_LED mnthl_

lght_typLED -0.520

mnthly_rnfl -0.059 0.115

traffic_db 0.076 -0.149 -0.045

**GLMM output with only *Scotophilus kuhlii* data**

Generalized linear mixed model fit by maximum likelihood (Laplace Approximation) ['glmerMod']

Family: Negative Binomial(1.326) ( log )

Formula: SCKU_passes ~ light_type + monthly_rainfall + traffic_db + (1 | site)

Data: bat_data

AIC BIC logLik deviance df.resid

571.1 582.6 -279.6 559.1 44

Scaled residuals:

Min 1Q Median 3Q Max

-1.1295 -0.6809 -0.3106 0.7032 2.4756

Random effects:

Groups Name Variance Std.Dev.

site (Intercept) 0.05794 0.2407

Number of obs: 50, groups: site, 5

Fixed effects:

Estimate Std. Error z value Pr(>|z|)

(Intercept) 4.51996 0.21070 21.452 < 2e-16 ***

light_typeLED 0.08409 0.25132 0.335 0.737937

monthly_rainfall 0.45397 0.12680 3.580 0.000343 ***

traffic_db 0.12699 0.15848 0.801 0.422946

---

Signif. codes: 0 ‘***’ 0.001 ‘**’ 0.01 ‘*’ 0.05 ‘.’ 0.1 ‘ ’ 1

Correlation of Fixed Effects:

(Intr) lg_LED mnthl_

lght_typLED -0.603

mnthly_rnfl -0.015 0.053

traffic_db 0.124 -0.134 0.014

**GLMM output with only *Saccolaimus saccolaimus* data**

Generalized linear mixed model fit by maximum likelihood (Laplace Approximation) ['glmerMod']

Family: Negative Binomial(1.3094) ( log )

Formula: SASA_passes ~ light_type + monthly_rainfall + traffic_db + (1 | site) + light_type:monthly_rainfall + light_type:traffic_db

Data: bat_data

AIC BIC logLik deviance df.resid

403.8 419.1 -193.9 387.8 42

Scaled residuals:

Min 1Q Median 3Q Max

-1.0676 -0.7432 -0.3158 0.3854 3.8979

Random effects:

Groups Name Variance Std.Dev.

site (Intercept) 0.4206 0.6485

Number of obs: 50, groups: site, 5

Fixed effects:

Estimate Std. Error z value Pr(>|z|)

(Intercept) 2.6864 0.3503 7.668 1.74e-14 ***

light_typeLED 0.2140 0.2861 0.748 0.454454

monthly_rainfall 0.6328 0.2147 2.947 0.003211 **

traffic_db 0.4490 0.2144 2.094 0.036242 *

light_typeLED:monthly_rainfall -0.8003 0.2991 -2.676 0.007461 **

light_typeLED:traffic_db -0.9934 0.2861 -3.472 0.000517 ***

---

Signif. codes: 0 ‘***’ 0.001 ‘**’ 0.01 ‘*’ 0.05 ‘.’ 0.1 ‘ ’ 1

Correlation of Fixed Effects:

(Intr) lg_LED mnthl_ trffc_ lght_typLED:m_

lght_typLED -0.413

mnthly_rnfl -0.022 0.034

traffic_db 0.093 -0.230 0.054

lght_typLED:m_ 0.033 -0.062 -0.720 -0.031

lght_typLED:t_ -0.049 0.041 -0.150 -0.488 0.268
