# Supplementary figures and images for: Ecological impacts of the LED-streetlight retrofit on insectivorous bats in Singapore
Source: PLoS One. 2021 May 26;16(5):e0247900. doi: 10.1371/journal.pone.0247900 (PMC8153503; doi:10.1371/journal.pone.0247900)

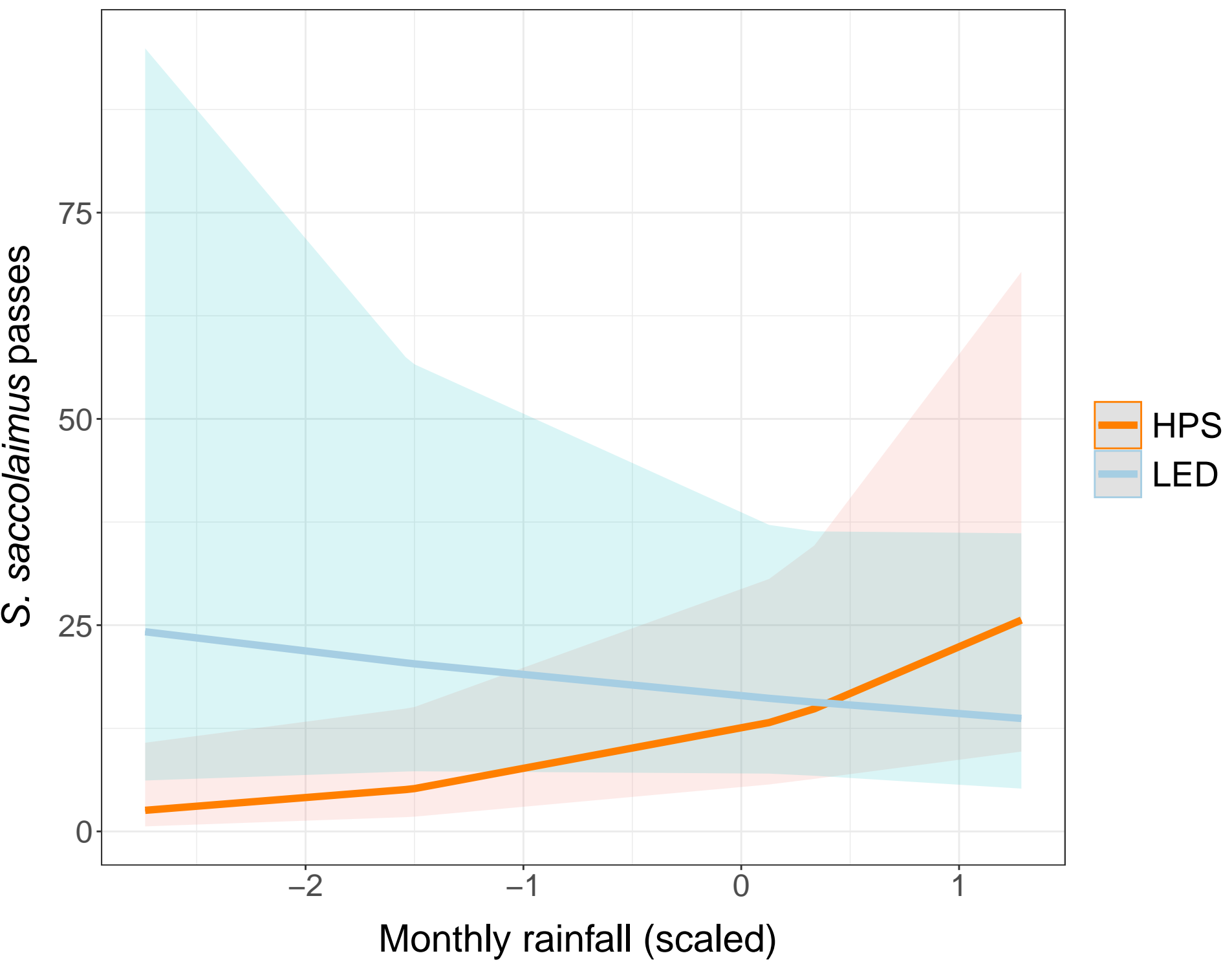

*S. saccolaimus* passes

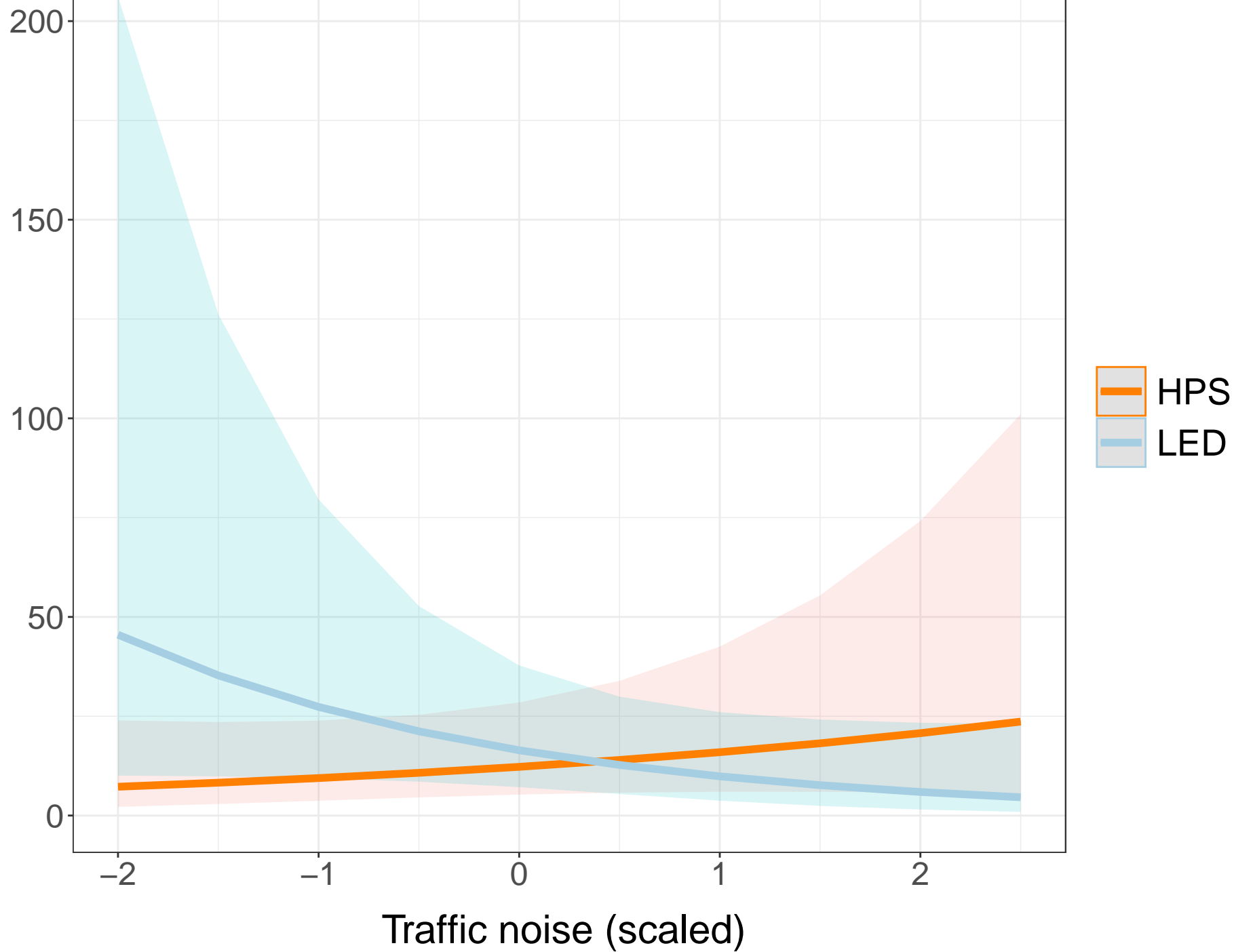

Supplement: S4 Appendix — (PDF) [file pone.0247900.s004.pdf]
