# Supplementary material for: Ecological impacts of the LED-streetlight retrofit on insectivorous bats in Singapore
Source: PLoS One. 2021 May 26;16(5):e0247900. doi: 10.1371/journal.pone.0247900 (PMC8153503; doi:10.1371/journal.pone.0247900)
Supplement: S5 Appendix — (PDF) [file pone.0247900.s005.pdf]

The Effect of an Experimental Light Treatment on Bats at an Urban Water Body

Michelle Gan Wan Jie
A0115644J

A thesis submitted to
Department Of Biological Sciences
National University Of Singapore
in partial fulfilment for the
Degree of Bachelor of Environmental Studies
with Honours in
Environmental Biology

Life Sciences Honours Cohort
AY2016/2017 S1

DECLARATION

I hereby declare that this thesis is my original work and it has been written by me in its entirety. I have duly acknowledged all the sources of information which have been used in the thesis.

This thesis has also not been submitted for any degree in any university previously.

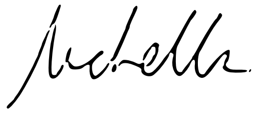

Name of Student
04 April 2017

ACKNOWLEDGMENTS

I would like to extend my heartfelt gratitude to my supervisor, Dr. Joanna Coleman, for her guidance and constant encouragment throughout this project. In addition, I would like to thank National Parks Board and SG Botanic Gardens (with special thanks to Mr. Elango Velautham, Deputy Director of Arboriculture and Plant Resources) for hosting me and allowing me to conduct my study on their premises.

To my fellow FYP “lab”mates, Deon Lum, Joan Lim, Loh Jia Xin and Nicolette Loo…we made it to the end! Thank you for patiently sitting through my rants and breakdowns and for all your feedback and help. I am glad we were in this together.

To my friends who accompanied me during my fieldwork, encouraged me when I felt down, nagged at me to not procrastinate and were always willing to offer a helping hand, thank you from the bottom of my heart. You know who you are.

And last but certainly not least; I would like to thank my family for the constant support and concern they have showered on me. Thank you for being ever so understanding during this period especially towards the end. I could not have done this without you.

TABLE OF CONTENTS

ACKNOWLEDGMENTS ..... **ii**

TABLE OF CONTENTS ..... **iii**

ABSTRACT ..... **iv**

1. INTRODUCTION **1**

1.1 Ecological impacts of artificial lighting 1

1.2 Impacts of artificial lighting on bats 2

1.3 Singapore's context 4

2. METHODS AND MATERIALS **7**

2.1 Study site 7

2.2 Experimental setup 7

2.3 Data processing 10

2.4 Species identification 11

2.5 Statistical analysis 13

3. RESULTS **15**

3.1 Species richness and diversity 15

3.2 Bat activity 16

3.3 Temporal activity patterns 19

4. DISCUSSION **22**

4.1 Species richness and diversity 22

4.2 Effect of light on bat activity 24

4.3 Limitations and future studies 28

5. CONCLUSION **30**

6. REFERENCES **31**

7. APPENDIX **38**

ABSTRACT

In the past century, the unprecedented rise in artificial lighting has led to the issue of light pollution. Numerous studies have documented the impacts of artificial lighting on biodiversity and ecosystems. Being nocturnal, bats are likely among species most affected by artificial light. However few studies have been done in the tropics and to date, none in Singapore. This a novel study that aims to determine the effects of an artificial light treatment on bats at an urban water body – important drinking and foraging habitats for bats. A light-emitting diode light was used to illuminate an urban water body. Bat species richness and diversity along with overall and temporal activity was compared between treatment and control nights (n=30) using acoustic monitoring. Three species and one genus was observed: *Scotophilus kuhlii*, *Saccolaimus saccolaimus*, *Myotis* spp. and Unknown (possibly *Pipistrellus stenopterus*). The artificial light was found to have no significant effect on all bats although generally, activity indices decreased on treatment nights except for *Myotis* spp. which exhibited the opposite trend. The foraging activity of *Scotophilus kuhlii* and Unknown was found to be affected by the light temporally. The results of this study can possibly be used to inform future lighting decisions.

1. INTRODUCTION

1.1 Ecological impacts of artificial lighting

Increasing global urbanisation, population growth, human development and advancements in lighting technology have contributed to the unprecedented rise in artificial lighting in the past century (Hölker et al., 2010a). Artificial lighting leads to the problem of light pollution, defined as the alternation of natural night light levels through artificial light sources (Falchi et al., 2011). This issue is of global concern, and although most prevalent in cities, even remote areas are affected (Falchi et al., 2016). In fact, artificial light is estimated to affect almost one-quarter of global land area (Cinzano et al., 2001; Falchi et al., 2016). With artificial light increasing by about 6% per year globally (Hölker et al., 2010b), light pollution is a growing problem. It is one we expect to increase even further in the future with increasing urbanisation and the advent of technology that is making lights cheaper and more efficient (Grimm et al., 2008; Schuber & Kim, 2008).

A growing body of research has documented impacts of artificial light on biodiversity and the alteration of ecosystems (Davies et al., 2013b; Gaston & Bennie, 2014; Hölker et al., 2010a, 2010b; Longcore & Rich, 2004). It is becoming increasingly clear that artificial light can affect both structural and functional diversity (Longcore & Rich, 2004; Gaston et al., 2015). As artificial light changes natural light distributions spatially and temporally, it disrupts the natural circadian cycles of many species (Gaston & Bennie, 2014). This, in turn, affects their physiology, behaviour and interactions with other species and the environment. As a result, light pollution is now considered a key biodiversity threat (Hölker et al., 2010b).

1.2 Impacts of artificial lighting on bats

Of all orders of mammals, bats are the second most speciose, and the most widely distributed (Voigt & Kingston, 2016). Given their unparalleled dietary diversity and voracious appetites, they also provide a large variety of ecosystem services. For example, as apex predators on nocturnal insects, insectivorous bats limit their populations, including species that are pests on commercially important plants. In the United States, the value of this ecosystem service to the agricultural industry was estimated at $23 billion USD per year (Boyles et al. 2011). Not only are they ecologically important, but bats are also very sensitive to habitat changes, with extremely slow life histories that make them inherently slow to recover from habitat changes (Mickleburgh et al., 2002; Jones et al., 2009). And so bats are effective bioindicators that can reflect the impacts of anthropogenic environmental change like the increasing presence of artificial light. Moreover, it stands to reason that these environmental changes, if they affect bats strongly enough, may have cascading consequences on entire ecosystems.

It is well established that bats are sensitive to artificial light. After all, they are nocturnal, and they have evolved an entire suite of physiological and behavioural adaptations specifically to the night (Voigt & Lewanzik, 2011). Artificial lighting affects natural light-dark cycles and thus impacts many bat behaviours and activity patterns. (Haeussler & Erkert, 1978). Roost emergence, for example, is highly influenced by the timing of sunset (Kunz, 1982). Bats whose roosts are illuminated by artificial light may thus have delayed emergence. This could easily lead to a mismatch between the time during with bats are foraging and the typical peak in insect abundance that occurs at sunset (Rydell et al., 1996). Artifical light can also affect connectivity of roosts to foraging habitats. For more light-adverse species, the presence of light along

their commuting route to foraging habitats can cause them to seek alternatives and failing that, even abandon their roosts (Stone et al., 2015a).

Studies have found that artificial light can enhance or compromise foraging opportunities for bats depending on the species (Stone et al., 2015a). Some are attracted to streetlights due to increased abundance of positively phototactic insects, such as moths (Rydell, 1992). The presence of large swarms of insects around some streetlamps, therefore, allows some bat species to forage more efficiently (Acharya & Fenton, 1999). But for others, artificial illumination of foraging habitats can depress foraging activity. For example, when foraging habitats were subject to partial illumination bats reacted by either flying quickly by or complete avoidance (Polak et al., 2011). Artificial light can also increase interspecific competition among bats as more light-sensitive species are unable to forage at illuminated foraging areas that more light tolerant species can exploit (Arlettaz et al., 2000). Artificial light has been found to change the community composition of insects (Davies et al., 2012), which may alter prey availability for light-sensitive species.

Artificial light might also affect the drinking behaviour of bats, although this area of research is extremely understudied. Drinking is an essential behaviour for most bats, including insectivorous species. Much water is lost from their increased body surface due to their wing membranes and respiratory system due to the demands of flight (Russo et al, 2012). They use diverse water bodies, including slowly-flowing rivers, ponds, lakes and artificial bodies of waters like reservoirs (Korine et al., 2016), all of which they may also use to forage on emerging aquatic insects (Ciechanowski, 2002). However, the illumination of freshwater bodies is becoming more prevalent, especially in cities, for safety or recreational purposes (Russo et al., 2017). The problem is that for bats to drink, they must swoop down to the water surface, and then lap up

water in flight (Korine et al., 2016). This requires them to decelerate and manoeuvre themselves very carefully – they have to get close enough to touch the water surface but not so close that they crash into it (Russo et al., 2016). This action could easily place bats at greater risk of predation, especially if they are drinking at an illuminated site (Russo et al., 2017). That bats perceive this as problematic is indicated by the finding that five of seven species (including some that had been observed to be light-tolerant) reduced their drinking activity when a water body was illuminated (Russo et al., 2017).

The effect of artificial light on bats is variable and seems dependent on foraging strategy and flight style. The most light-sensitive species tend to be those that forage in cluttered environments and are, therefore, slow-flying and more manoeuvrable (Stone et al., 2009, 2012). Such bats may therefore have a greater perceived risk of predation of illuminated areas (Jones & Rydell, 1994). Contrastingly, species that hunt in the open are usually faster fliers and tend to be more light-tolerant and able to exploit illuminated foraging areas (Rydell, 1992). But even they can be negatively affected by artificial light in cities that reduce their abilities to cross gaps (Hale et al., 2015).

1.3 Singapore's context

While there has been a growing body of research on the effect of artificial light on bats, a disproportionate number of studies have been conducted in temperate areas. Very few have been done in the tropics, and there are none from Southeast Asia, whose bat fauna accounts for more than one-quarter of all bats worldwide (Kingston, 2010).

Singapore, recently named the most light polluted city in the world (Falchi et al, 2016), presents a very interesting location to study the effects of artificial light on bats. Despite being completely urbanised and having lost more than 95% of its original forest cover, Singapore has increased its green cover to 46.5% through intensive greening

efforts (Brook et al., 2003; Chin, 2008). In addition, it is unique among tropical nations in its lack of natural freshwater resources – a problem it has circumvented by building water catchment areas. Studies have shown that even in cities, mosaic landscapes consisting of urban and green spaces with water bodies can support high bat species richness and diversity (Hourigan et al., 2010; Coleman, 2011; Fabianek et al., 2011).

There are currently 19 confirmed microbat species present in Singapore which can be found in a variety of habitats ranging from forests to urbanised areas (Pottie et al., 2005; Lane et al., 2006; Chan et al., 2009; Leong & Lim, 2009; Leong et al., 2010 Lim & Leong, 2014). The foraging styles of these bats vary from fast-flying in open spaces to slow-flying in cluttered environments like forests.

There have been very few studies on bats in Singapore and none on the effects of artificial light on its bats. I was interested in how artificial light affects bats at water bodies because the only study that has thus far investigated this question (Russo et al., 2017) found a reduction in foraging and drinking activity. However, that study was done in Italy, which has a Mediterranean climate, and I wondered how applicable its findings might be to bats in wetter climates. I therefore conducted an experiment to test the effect of an artificial light treatment on bats at an urban water body. My research objectives were to determine whether:

1. artificial light affects species richness and diversity
2. bat activity is influenced by the presence of light
3. artificial light influences the temporal distribution of bat activity

I hypothesise that artificial light has a negative impact on bats and that this effect is especially noticeable at a water body because vulnerability to predation should increase in open areas. Therefore, I predicted that diversity, abundance, and foraging activity of

bats all decrease under artificial illumination. Furthermore, I hypothesise that artificial lights most negatively affect slow-flying species that are adapted to forage in cluttered microhabitats because they are inherently more vulnerable to predation. Therefore, I predicted that the above-mentioned predicted impacts are more strongly observed in these species than in faster-flying species that are adapted to using open microhabitats.

2. METHODS AND MATERIALS

2.1 Study site

I conducted my study at a 0.65 hectare pond called Eco-lake in the Singapore Botanic Gardens (SBG) (Google Earth Pro, 2017). The SBG, encompassing 74 hectares, contains a mosaic of habitats like water bodies, primary rainforests, lawns and gardens, therefore allowing it to support a wide diversity of fauna (National Parks, 2014). It is a refuge for wildlife in the overall area, which has undergone extensive urban development (National Parks, 2014). The SBG provides bats with many potential foraging and roosting habitats. Although the exact number of bat roosts in the SBG is unknown, several bat species have been previously recorded to roost and forage at the ponds in the area (Pottie et al., 2005; J. Coleman, pers. comm.).

Of the three large ponds in the SBG, I chose Eco-lake because it had the most ideal conditions for my study. It is the darkest, and has the lowest amount of surface vegetation but the greatest shoreline vegetation – these conditions are known to promote bat activity (Boonman et al., 1998; Ciechanowski, 2002). Eco-lake also has much less human traffic and noise after sunset compared to the other two ponds.

2.2 Experimental setup

From October to December 2016, I conducted experimental trials at a single location, which was as far as possible from any pathways (Fig.1). This allowed me to minimize the influence of ambient light and human disturbance.

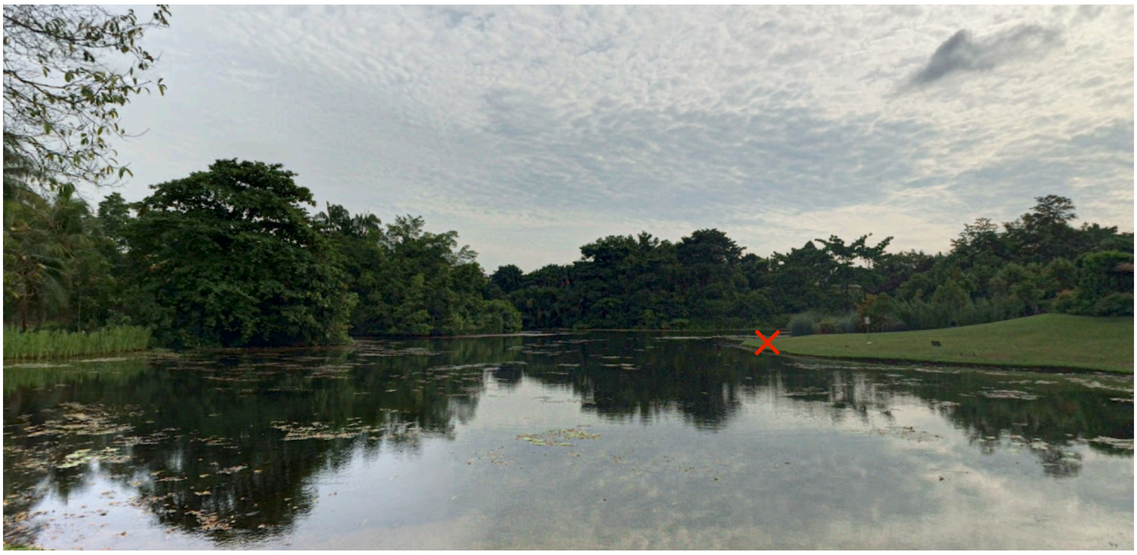

Figure 1: Experimental location (Red X) at the Eco-lake, SBG. The experimental location was 40m away from the nearest pathway.

On control nights, I had no source of artificial light. On treatment nights, I used a portable, rechargeable light emitting diode (LED) light of the type that is commonly used for artificial illumination outdoors. Its 40 LEDs generate a white light, with an intensity of 1800 lumens (5800K) and have a power rating of 20W. I placed it on a stand, at a height of 1.7m above the ground, and oriented it to face the pond (Fig. 2). I referenced the light levels of path lights in the SBG to ensure that those of my LED light treatment were of a similar range. It might have been more appropriate to use high-pressure sodium-vapour (HPSV) lamps, like most of the lights in the SBG but those can only run on a generator, making them impractical for my study. And one advantage to LEDs is that they are increasingly being implemented in cities to replace HPSV lighting, including in Singapore (Abdullah, 2017).

To measure bat activity, I recorded bat echolocation calls with an ultrasound microphone (M500 USB, Pettersson Elektronik AB, Sweden, 2014), oriented toward the light and upward at a 45° angle. The microphone's directional horn ensured that it would only detect echolocation calls emitted by bats flying within the illumination path of the light (Fig. 2). I connected the microphone to a tablet (Acer Iconia W510-1422),

and used BatMic software (Pettersson Elektronik 2014) to save recordings as 1 minute .WAV files. As only microbats echolocate, my experiment design could only measure the activity of microbats.

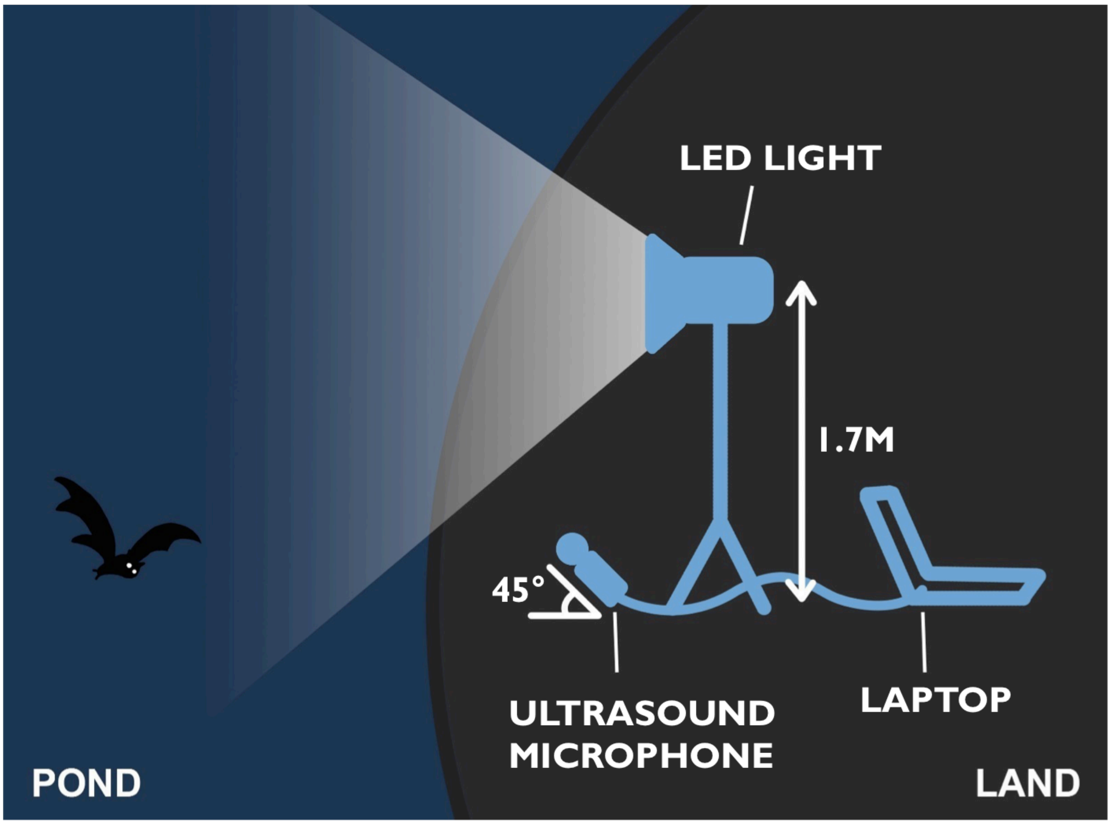

Figure 2: Diagram of the experimental set-up.

I began each trial at sunset (usually when the time of peak insect emergence occurs and hence peak bat activity; e.g., Rydell et al., 1996; Fukui et al., 2006), and recorded bats for one hour. In total, I had 30 trials: with 15 treatment and 15 control nights, which I alternated. I only carried out trials on nights with favourable weather conditions (wind speeds < 10kph; no rain). At the start of every session, I recorded environmental variables, including ambient temperature, wind speed, moon phase and ambient light. At the end of each session, I recorded ambient light again to obtain a better measure of the interaction between moon phase and cloud cover.

2.3 Data processing

I analysed 30 hours of acoustic recordings using Kaleidoscope Viewer (v4.0.2, Wildlife Acoustics Inc., Massachusetts, USA), which allows echolocation calls to be viewed as a spectrogram. When bats echolocate, their calls can be categorised into: search phase, approach phase and terminal buzz (Fig. 3). Bats emit search phase calls for general navigation and to scan for prey (Griffin et al., 1960). Search phase calls are best used for species identification as they usually have consistent call characteristics and are well separated in time (Griffin et al., 1960). Approach phase calls are emitted when approaching prey and terminal buzz calls when in pursuit or capture of prey.

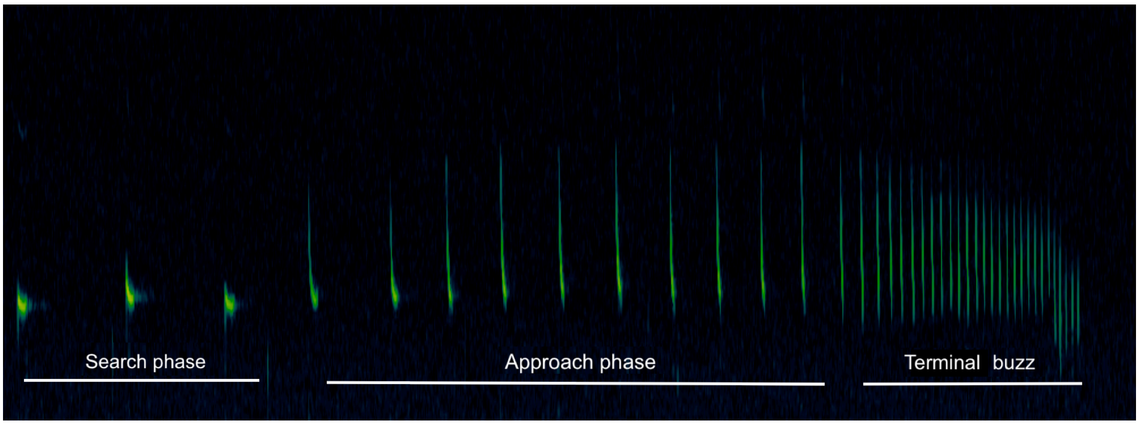

Figure 3: Spectrogram of a bat pass with terminal buzz

Although bat detectors can effectively record echolocation calls, it is not possible to distinguish whether an individual bat made each call, and so they only allow activity to be quantified – bat researchers then use this as an index (but not a direct measure) of abundance (Jung & Kalko, 2010). I specifically measured three bat activity indices: the number of bat passes, the number of terminal buzzes and buzz ratio (terminal buzzes per pass).

I defined a bat pass as two or more echolocation calls separated from subsequent passes by one second of silence (Thomas, 1988). Terminal buzzes are indicative of a foraging or drinking attempt by a bat and occur whether the attack is successful or not

(Ratcliffe, 2013). It is distinguished from other calls by a rapid increase in the repetition rate and decreasing frequency (Schoeman, 2015; Fig. 3). The buzz ratio (terminal buzzes per pass) is a measure of the intensity of foraging that controls for overall bat activity. It can also be used as a proxy of bat prey abundance - the higher the ratio, the more insects that bats prey on likely to be present (Rowse et al., 2016a). As a measure of bat prey, buzz ratio may be as good or better than insect sampling which can be biased as light traps only attract some insects that bats prey on and bats may not be flying where sticky traps are usually placed (R. Barclay, pers. comm.).

2.4 Species identification

The existence of comprehensive reference call libraries for bat assemblages in many temperate-zone regions, e.g., North America, has made it possible to develop software that automates the task of identifying echolocation calls to species (Schober, 1997). Such libraries are constructed by capturing sufficiently large numbers of individuals of each species, and then recording them after hand-release in the habitat(s) under study. Unfortunately, no such libraries exist for bats in Singapore. I therefore partly based my species identifications on the information presented by Pottie et al. (2005), who surveyed bats in a small, but unspecified subset of habitats in Singapore. However, the applicability of the echolocation call data is questionable because: (1) there is no specific information on sites at which bats were recorded; (2) an unspecified number of reference calls were obtained from individuals released into an aviary (which is not an appropriate method, J Coleman, pers. comm.); and (3) numbers of individuals sampled were unacceptably low for some species (J Coleman, pers. comm.) Given these limitations, I also supplemented my echolocation call reference material from papers from around the region and used them as references for species identification (See Appendix A).

I used search phase calls to identify bats to species where possible. Of four different call types that I recorded, I could identify two to species, one to genus and could not identify the fourth one (Fig. 4).

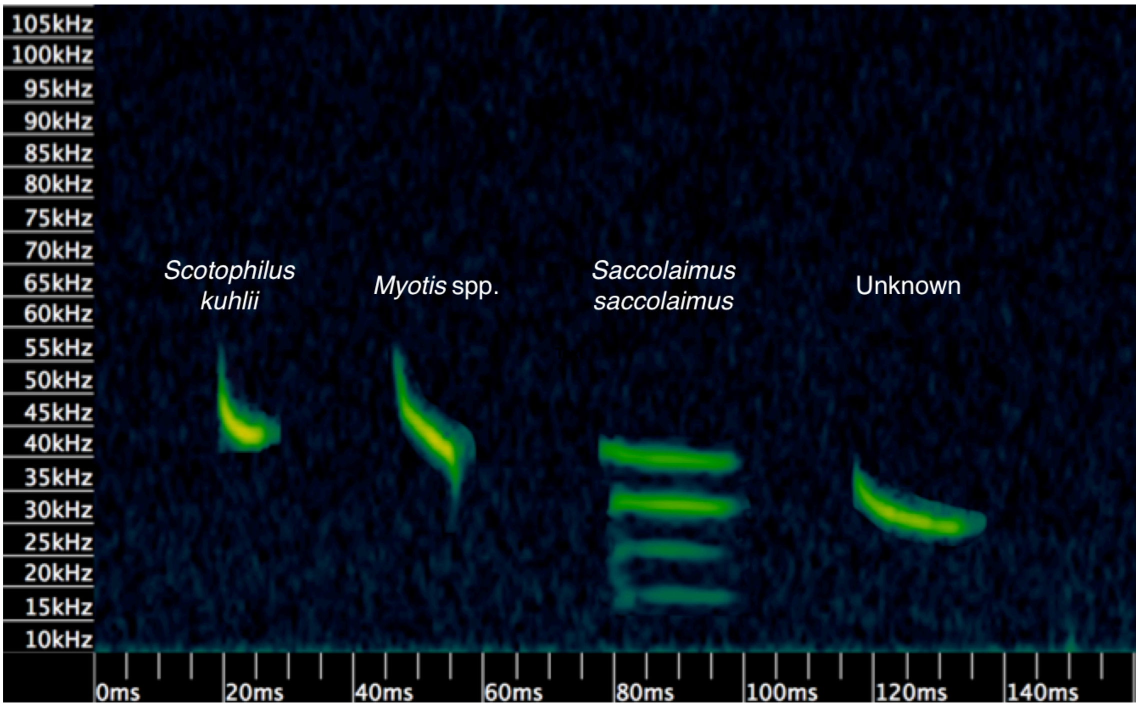

Figure 4: Spectrogram of search phase calls of bats observed.

Most *Myotis* bats are notoriously difficult to differentiate on the basis of echolocation calls, so I only identified them to genus (Russ, 2012). Three *Myotis* species have been recorded in Singapore: *M. adversus*, *M. horsfieldii* and *M. muricola*. But the calls of *M. muricola* are sufficiently distinct that they are unlikely to be confused with those of its congeners (Pottie et al., 2005). Therefore, the *Myotis* bats I recorded are more likely to be *M. adversus* or *M. horsfieldii*, both of which are also highly associated with water bodies (Pottie et al., 2005; Tan & Lim, 2014).

Call parameters for the unknown species did not match any of those listed in Pottie et al. (2005), but further research suggests that they seem consistent with those of *Pipistrellus stenopterus* (Kingston et al., 2003). This species has been observed in Singapore in and around the SBG (Leong et al., 2010). But a lack of published records

led Pottie et al. (2005) to label it as unknown. Because the last confirmed encounter with this bat was in 2010 and no confirmed roosting sites have been found in Singapore, I opted not to conclusively label my unknown bat as *P. stenopterus*.

2.5 Statistical analysis

I used the mean number of passes per night to evaluate the impact of the light treatment on species richness and diversity. Species richness was simply the total number of species observed. For species diversity, I used the Shannon-Wiener index (H) and the Berger-Parker index (D), which measures dominance independently of species richness (Magurran, 2004).

I assessed the effect of the light treatment on all three bat activity indices (number of bat passes, total buzzes, buzz ratio) for all species and each species / species group separately, with a generalized linear model (GLM) in R studio (Version 1.0.136). I included variables known to influence bat activity, i.e., maximum temperature, wind speed at sunset and ambient light levels (measured at end of the session). I checked all explanatory variables for multicollinearity using the VIF() function in the R package 'car', and found none. I started out with a saturated model and removed non-significant terms sequentially. Response variables were counts and were not normally distributed. Therefore, to determine the best error distribution, I tested two full factorial GLMs for each species and activity index, one with a Poisson and the other with a negative binomial distribution. I compared model fit using Akaike's Information Criterion (AIC) and a goodness-of-fit test. Negative binomial distributions consistently provided better fit (Appendix B).

To test the effect of the light treatment on the temporal distribution of bat activity, I grouped bat activity data into 10-minute intervals for a total of six intervals. I

used GLMs to compare treatment and control nights for each interval. I determined VIF and model fit as above. Because I did not record enough terminal buzzes for *S. saccolaimus*, I did not analyse the temporal distribution of this species' activity. I used R studio (Version 1.0.136) for all analyses.

3. RESULTS

I recorded a total of 15,184 passes and 11,534 terminal buzzes from four species or species groups (Table 1). I identified 70.61% of passes to species, and 5.27% of passes to genus level for *Myotis* calls. 24.1% of all bat passes belonged to Unknown, a species I could not conclusively identify. The most commonly observed bat was *S. kuhli* and *Myotis* spp. was the least common.

Table 1: Total numbers of bat passes and terminal buzzes for each species or species group recorded for two experimental treatments –lit and control. Do note that Myotis spp. was only observed for five treatment (lit) nights and eight control nights.

Species	Bat passes			Terminal buzzes		
	Lit	Control	Total	Lit	Control	Total
Scotophilus kuhlii	4858	4904	9762	4332	4468	8800
Unknown	1698	1964	3662	380	696	1076
Saccolaimus saccolaimus	456	504	960	46	116	162
Myotis spp.	332	468	800	804	692	1496
Overall	7344	7804	15,184	5562	5972	11,534

3.1 Species richness and diversity

Species richness did not differ between the control and treatment nights (Table 2). However, I only recorded *Myotis* spp. on five treatment nights and eight control nights, whereas I recorded the other three bats every night. The diversity indices shown did not differ between treatment and control nights (Table 2).

Table 2: Species richness, Berger-Parker Index and Shannon-Wiener Index for control and treatment nights.

	Species richness	Berger-Parker Index	Shannon-Wiener Index
Control	4	0.63	0.56
Lit	4	0.66	0.52

3.2 Bat activity

For the entire bat assemblage, I detected no difference in any activity index between treatment and control nights (Table 3; Fig. 5).

The light treatment also had no detectable effect on total bat passes, terminal buzzes and buzz ratio for *S. kuhlii*, Unknown, *S. saccolaimus* and *Myotis* spp (Table 3). In general, activity indices were lower (but not significantly) on treatment nights for all four species/species groups except *Myotis* spp., which seemed to exhibit the opposite trend (Table 1, Figs. 6-9). Ambient light was found to have a significant negative effect on Unknown's bat passes and Myotis spp. bat passes and buzzes.

Table 3: Effect of the light treatment for each activity index of all species and each species or species group. Environmental variables that were found to have a significant effect are also listed in this table. (See Appendix C for all other variables)

Species	Variable	Activity index	Estimated effect (β ± SE)	p-value
Overall	Light	Bat pass	-0.03±0.06	0.60
		Terminal buzz	-0.17±0.26	0.53
		Buzz ratio	-0.13±0.47	0.78
S. kuhlii	Light	Bat pass	$-1.54e^{-02} \pm 7.89e^{-02}$	0.85
		Terminal buzz	-0.25±0.31	0.43
		Buzz ratio	-0.14±0.44	0.74
Unknown	Light	Bat pass	-0.15±0.09	0.12
		Terminal buzz	-0.61±0.33	0.07
		Buzz ratio	-0.31±0.79	0.69
	Ambient light	Bat pass	-1.63±0.48	**<0.01**
S. saccolaimus	Light	Bat pass	-0.15±0.09	0.12
		Terminal buzz	-0.93±0.55	0.09
		Buzz ratio	-0.58±1.01	0.56
Myotis spp.	Light	Bat pass	1.74±1.05	0.09
		Terminal buzz	1.74±1.25	0.16
		Buzz ratio	0.10±0.61	0.88
	Ambient light	Bat pass	-3.75±4.23	**0.02**
		Terminal buzz	-1.48±0.33	**0.02**

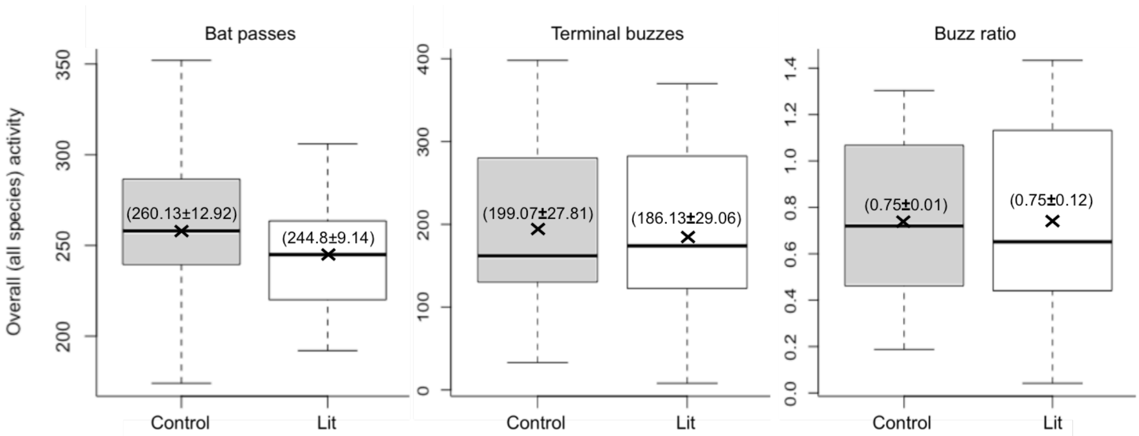

Figure 5: Comparison of activity indices for all species on lit and control nights. X denotes mean (± SE) number of passes, buzzes and buzz ratio per observed night. Overall species activity tended to be higher or the same on control nights, but the light treatment had no significant effect on any activity indices.

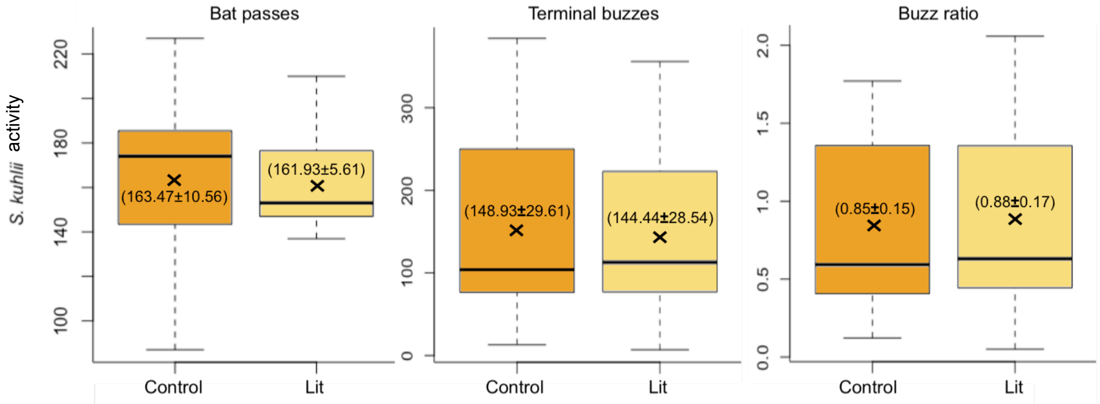

Figure 6: Comparison of activity indices for *S. kuhlii* on lit and control nights. X denotes mean (± SE) number of passes, buzzes and buzz ratio per observed night. *S. kuhlii* activity tended to be marginally higher on control nights but the light treatment had no significant effect on any activity indices.

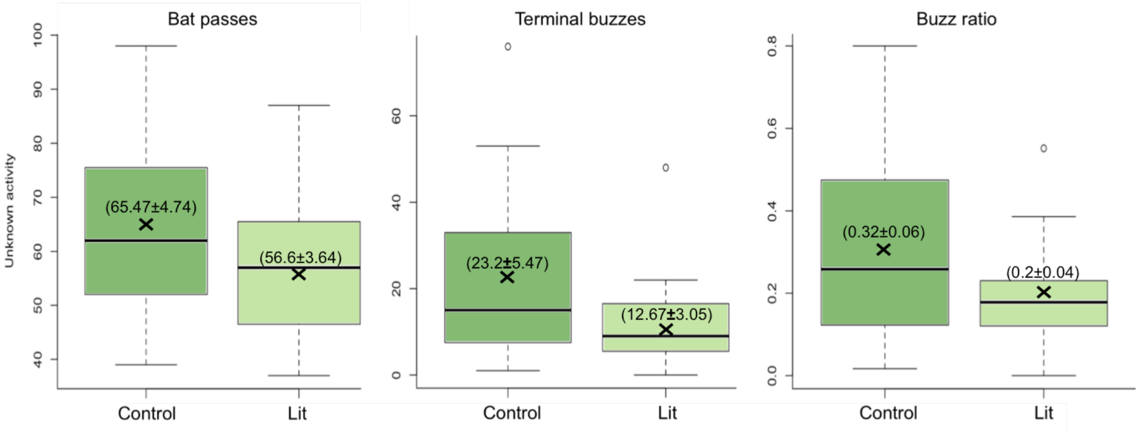

Figure 7: Comparison of activity indices for Unknown on lit and control nights. X denotes mean (± SE) number of passes, buzzes and buzz ratio per observed night. Unknown activity tended to be higher on control nights but the light treatment had no significant effect on any activity indices.

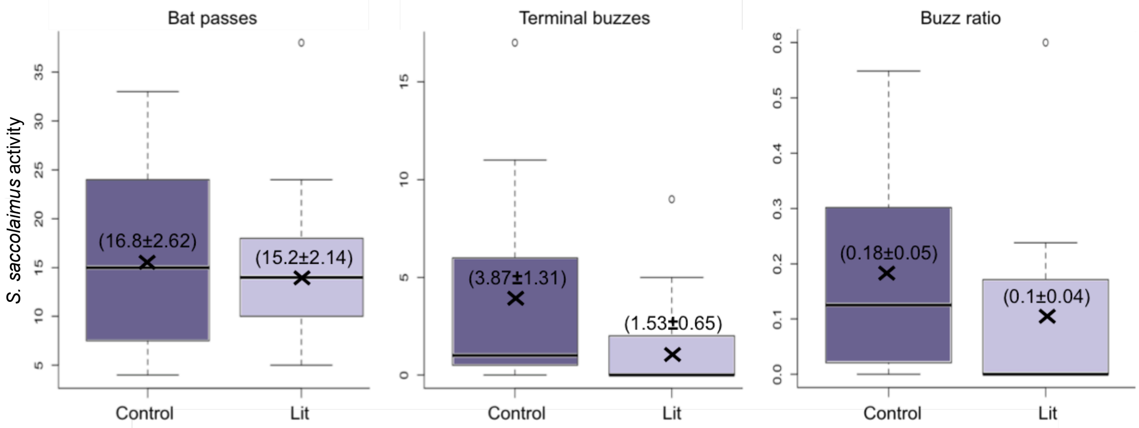

Figure 8: Comparison of activity indices for *S. saccolaimus* on lit and control nights. X denotes mean (± SE) number of passes, buzzes and buzz ratio per observed night. *S. saccolaimus* activity tended to be higher on control nights but the light treatment had no significant effect on any activity indices.

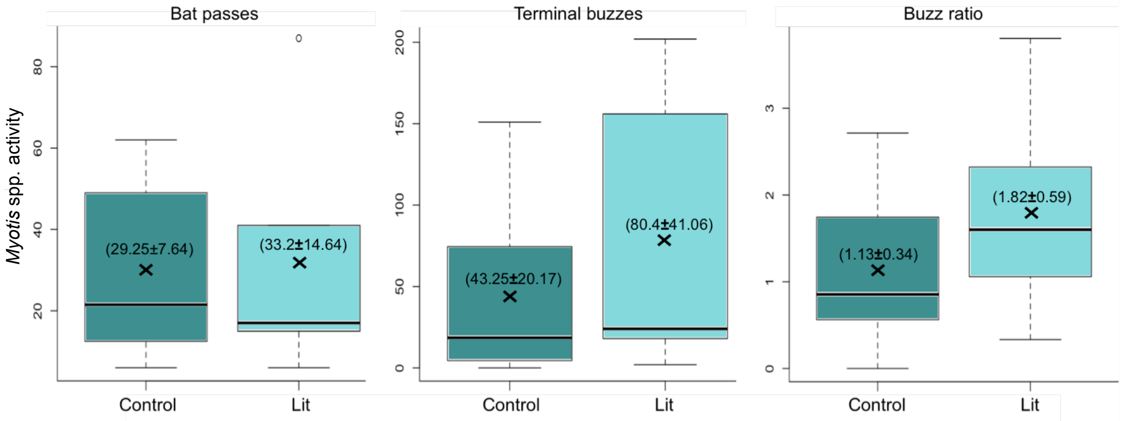

Figure 9: Comparison of activity indices for *Myotis* spp. on lit and control nights. X denotes mean (± SE) number of passes, buzzes and buzz ratio per observed night. *Myotis* spp. activity tended to be higher on light treatment nights but the light treatment had no significant effect on any activity indices.

3.3 Temporal activity patterns

The light treatment had no effect on activity indices for the entire bat assemblage, *Myotis* spp and *S. saccolaimus*. However, it did alter the temporal distribution of buzzes and buzz ratio for *S. kuhlii* and Unknown (Table 4).

Table 4: The effect of the light treatment for each time interval. *S. kuhlii* buzzes and buzz ratio were significantly higher 30-60 minutes after sunset on treatment nights. Unknown buzzes and buzz ratio were significantly higher in the first and fourth interval.

Species	Activity index	Interval (minutes after sunset)	Estimated effect of light (β ± SE)	p-value
S. kuhlii	Bat pass	1 (0-10)	0.04±0.19	0.86
		2 (10-20)	-0.04±0.11	0.73
		3 (20-30)	-0.04±0.10	0.68
		4 (30-40)	0.02±0.07	0.74
		5 (40-50)	0.03±0.08	0.68
		6 (50-60)	-0.07±0.08	0.37
	Terminal buzz	1 (0-10)	-0.84±0.47	0.07
		2 (10-20)	-0.18±0.39	0.64
		3 (20-30)	-0.09±0.33	0.78
		4 (30-40)	0.45±0.21	**0.03**
		5 (40-50)	0.51±0.18	**<0.01**
		6 (50-60)	0.46±0.18	**0.01**
	Buzz ratio	1 (0-10)	-0.29±0.42	0.50
		2 (10-20)	-0.09±0.33	0.78
		3 (20-30)	-0.01±0.34	0.98
		4 (30-40)	0.35±0.41	**0.02**
		5 (40-50)	0.25±0.18	**<0.01**
		6 (50-60)	0.27±0.21	**0.01**
Unknown	Bat pass	1 (0-10)	-0.60±0.34	0.08
		2 (10-20)	-0.04±0.23	0.87
		3 (20-30)	-0.23±0.25	0.36
		4 (30-40)	-0.03±0.15	0.82
		5 (40-50)	-0.13±0.15	0.38
		6 (50-60)	-0.08±0.60	0.62
	Terminal buzz	1 (0-10)	-1.99± 0.88	**0.02**
		2 (10-20)	-0.37±0.64	0.56
		3 (20-30)	-0.94±0.55	0.09
		4 (30-40)	-1.18±0.50	**0.02**
		5 (40-50)	-0.06±0.45	0.89
		6 (50-60)	-0.14± 0.42	0.74
	Buzz ratio	1 (0-10)	-1.11±0.47	**0.02**
		2 (10-20)	-0.33±0.67	0.62
		3 (20-30)	-0.73±0.37	0.39
		4 (30-40)	-1.52±0.55	**0.02**
		5 (40-50)	-0.17±0.91	0.86
		6 (50-60)	-0.19±0.93	0.84

Buzzes of *S. kuhlii* seemed to peak 10-20 minutes after sunset (Fig. 10), but its activity was relatively constant over each night. The temporal distribution of bat passes did not differ between treatment and control nights, but that of its foraging activity certainly did. The trend for *S. kuhlii* was that on treatment nights, buzzes tended to be lower (but not significantly) in the first 0-30 minutes after sunset before becoming higher 30-60 minutes after sunset (Fig. 10, Table 4).

Passes by the unknown species seemed to continually increase in the hour after sunset before peaking at 30-40 minutes (Fig. 11). The temporal distribution of passes did not differ between treatment and control nights. Buzzes of this bat peaked 20-30 minutes after sunset on both treatment and control nights. On treatment nights, I observed a reduction in buzzes in the first and fourth 10-minute-intervals after sunset relative to control nights (Fig. 11, Table 4).

In summary, I observed that the general trend was that, for all bats the light treatment seemed to either depress activity or have no effect, and did not enhance activity except for *Myotis* spp. However, none of these trends were significant based on my models. The light treatment also only affected the temporal foraging activity of *S. kuhlii* and Unknown.

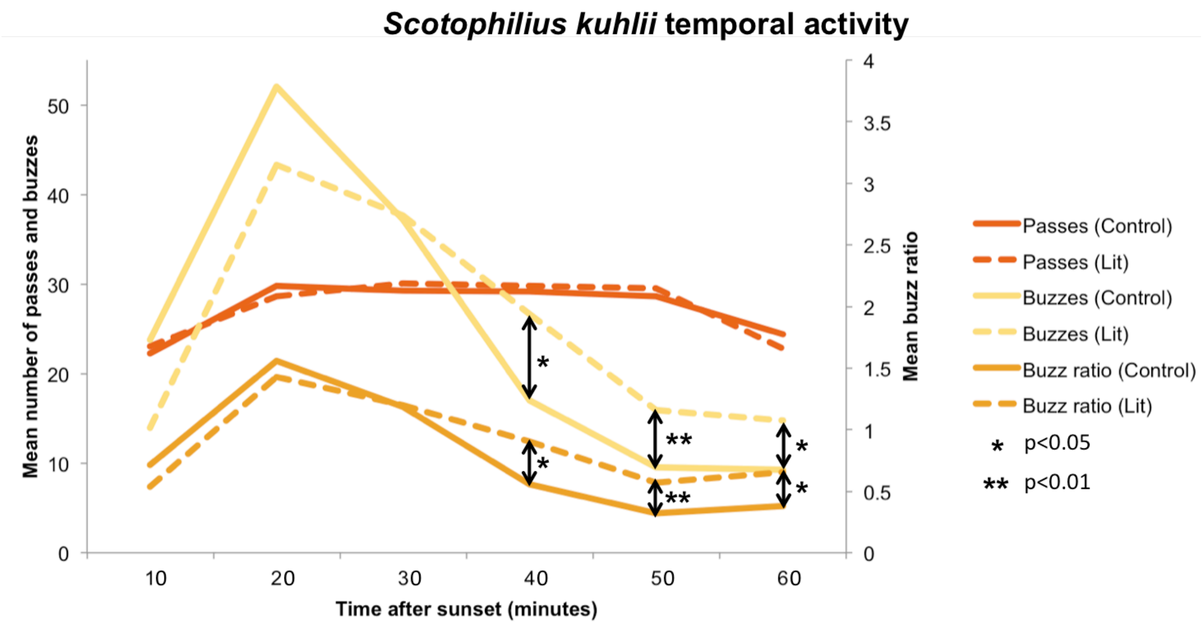

Figure 10: The temporal activity pattern of *S. kuhlii* for the hour after sunset. Activity indices shown: mean number of bat passes, mean number of terminal buzzes and buzz ratio.

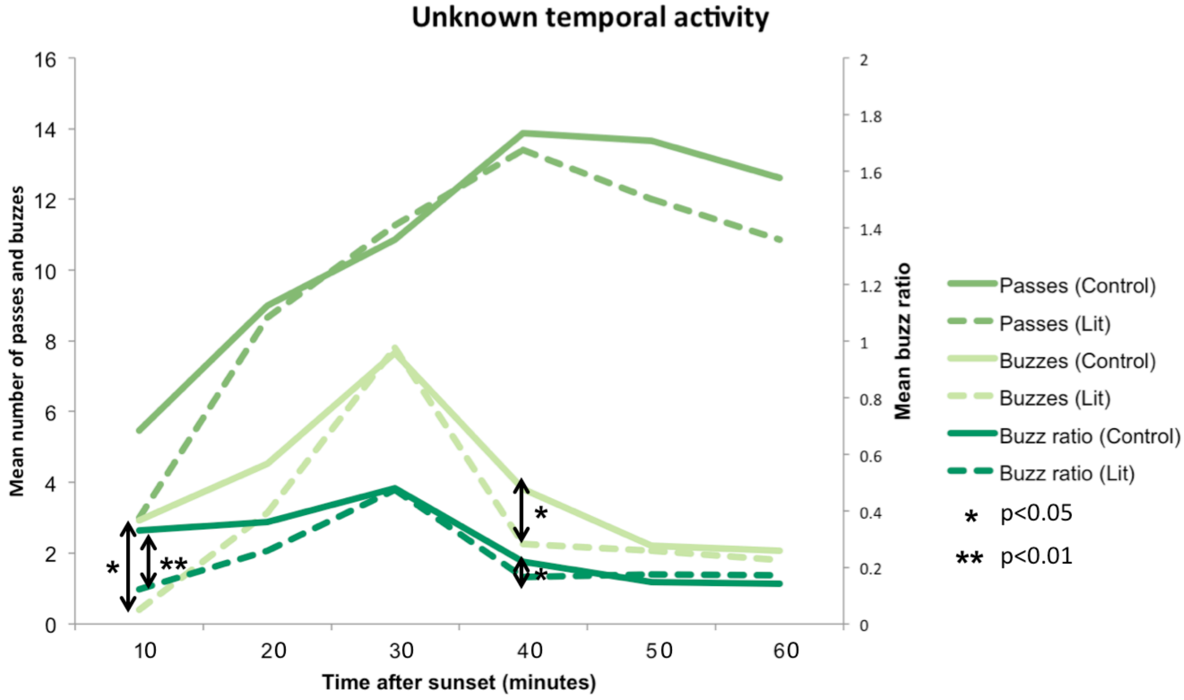

Figure 11: The temporal activity pattern of Unknown for the hour after sunset. Activity indices shown: mean number of bat passes, mean number of terminal buzzes and buzz ratio.

4. DISCUSSION

Little is known about the effects of artificial light on bats at water bodies, not just in Singapore but worldwide, and my study was the first to examine this question anywhere in the tropics. My results suggest no detrimental effect on bats generally, but an alteration to the temporal distribution of foraging activity of some species.

4.1 Species richness and diversity

I predicted that artificial light reduces the diversity of bats, but found no difference in species richness and diversity between treatment and control nights. Not only does this finding not support my hypothesis, but also it contrasts with results from other studies in which species richness and diversity tend be higher where and when it is darker (Scanlon & Petit, 2009; Linley, 2016; Russo et al., 2017). One possible explanation for my finding is that all observed species are considered common in Singapore, and are probably quite widespread around the island (Pottie et al, 2005). Therefore the probability of observing them would be higher as compared to a rless common species. Moreover, if the species I labelled 'unknown' really is *Pipistrellus stenopterus*, then this may suggest the need to revise its status from being indeterminate in Singapore, because it was the second most commonly observed bat in my study.

Because the same four species were present during both treatment and control nights, this might suggest that truly light-averse species like those of the genera *Rhinolophus* and *Hipposideros* that forage in cluttered environments might not even forage or drink at the Eco-lake. Although the Singapore Botanic Gardens and Eco-lake are already darker than most areas in Singapore (Robert, 2016), light-averse bats may not have a suitable commuting path from their roosts to Eco-lake because of the lit

pathways and open fields in the SBG, which might increase the perceived risk of predation (Stone et al. 2009, 2012). Similar studies looking at the effects of a change in light type also observed few to no light-averse bats although such bats were present in the study site (Stone et al., 2015b; Rowse et al., 2016a). It is also possible that the observed lack of relatively more rare light-adverse species in my study is an artefact of my small sample size. I had a total of 30 nights, which, according to Skalak et al. (2012), might only allow 80-90% of species occurring at a location to be sampled.

Out of all four species detected at Eco-lake, *Myotis* spp. was the only one not present every night. I only observed it on 13 nights. The Myotis bats that I likely observed are either *M. adversus* or *M. horsfieldii*. Both bats are highly associated with water bodies, because they forage by trawling, or flying above the water and catching prey items when their echolocation calls allow them to detect ripples on the surface (Denzinger & Schnitzler, 2013). This foraging style requires slow, maneuverable flight (Jones & Rayner, 1991). While perhaps not as light-adverse as bats that forage in cluttered microhabitats, *Myotis* bats might still be sensitive to light if it affects their ability to commute from their roosts to a water body. Many studies have observed a similar trend (Kuijper et al., 2008; Stone et al. 2009, 2012, 2015b; Rowse et al., 2016a), although none were conducted on tropical *Myotis* bats. *Myotis* bats also generally emit lower intensity calls, which could reduce their detectability by the ultrasound microphone (Bell, 1980; Faure et al., 1993; O'Farrell & Gannon, 1999). However, the intensity levels of *Myotis* bats observed in Singapore are not known and this might be a generalisation. It is also possible that as my study site only occupied part of Eco-lake, *Myotis* bats were simply foraging in another area with denser shore vegetation that increased prey abundance.

4.2 Effect of light on bat activity

4.2.1 Overall bat activity

I expected artificial light to reduce the abundance of bats – here (as in many other studies) I used activity as a proxy for abundance. However, I found no detectable effect of my light treatment on activity of the entire bat assemblage or individual species. Given that bats exhibit species-specific responses to light (Rydell, 1991), it is more informative to discern the effects of light on bat activity by discussing each species individually.

4.2.2 *Scotophilus kuhlii*

Scotophilus kuhlii is an aerial-hawking bat, which means that it pursues and catches its prey in flight (Zhu et al., 2012; Denzinger & Schnitzler, 2013). Bats with this foraging style are usually fast-flying and forage in open spaces. In Singapore, *S. kuhlli* is one of the most common microbats, and has been observed in many habitats although primarily in urban parklands and rural habitats (Pottie et al., 2005). It has been observed foraging at streetlights (e.g., Zhu et al., 2012). Therefore, either this species should have been less negatively affected by the light treatment compared to more slowly-flying species, or maybe even positively affected. However, I observed no effect. This could reflect my choice of light. The type of light and its spectral characteristics strongly influence its attractiveness to insects (Blake et al., 1994). In one study of the effects of LED lights on bats (Stone et al., 2012), species known to be light-tolerant that had previously been observed foraging at high pressure mercury vapour (HPMV) lamps showed no increased feeding activity at LED lights. LED lights in general, emit less ultraviolet light, which makes them less attractive to insects (Davies et al., 2013; Wakefield et al., 2016). The lack of an observable effect could also be attributed to the

short experimental nature of my study as it was only conducted for an hour each night and not consistently. Most bats that exploit streetlights as a foraging source do so as it is a predictable and permanent foraging habitat (Geggie & Fenton, 1985; Rydell, 1992). Therefore, bats in the area may not have had the time to recognise the light treatment as a forgaing source. The light that I used was also only placed at a height of 1.7m, which is lower than most path lights and streetlights in SGB and Singapore respectively. It is possible that since *S.kuhlii* forages high in open spaces, the light was at a height too low for it to forage at.

However, the temporal distribution of foraging activity of *S. kuhlii* was affected. More specifically, foraging activity increased in 30-60 minutes after sunset on treatment nights. Although the number of attacks (as indexed by buzzes) is not reflective of successful captures, an increase indicates increased foraging effort; especially considering no increase in passes (i.e., an index of abundance). What is unknown is whether *S. kuhlii* is opportunistically exploiting insects that are attracted to the light in the second half hour after sunset, when it is darker or whether its hunting success (proportion of successful attacks) is declining.

4.2.3 Unknown (possibly *Pipistrellus stenopterus*)

If the bat I labelled as 'unknown' really is *Pipistrellus stenopterus*, then it is a fast-flying aerial hawker (Kingston et al., 2003). In that case, I would expect it to respond similarly to *S. kuhlii* to my light treatment. However, that is not what I found (light had no discernable effect although the general trend was negative). Instead, ambient light negatively affected its activity and foraging activity. Why might the artificial light treatment have no effect, but ambient light have a negative one? *Pipistrellus* bats tend to be rather light-tolerant and some species exploit artificial light sources elsewhere in the world (Blake et al., 1994; Haffner & Stutz, 1985; Arlettaz et

al., 2000; Stone et al., 2009, 2012). However, even closely related species that are ecologically similar can exhibit differing tolerances to anthropogenic changes in the environment (Rowse et al., 2016b). It is possible *Pipistrellus stenopterus* in Singapore may not be as light-tolerant as its counterparts elsewhere in the world and this could be how it has been overlooked and eluded detection for many years. Perhaps, as open-air foragers (Leong & Chan, 2013), higher ambient light (e.g., as observed under higher moonlight levels and lower cloud cover) may increase the perceived risk of predation (Scanlon & Petit, 2008). Indeed, *Pipistrellus pipistrellus* (a known light-tolerant species) bats were found to be reluctant to fly under lit conditions in a laboratory (Speakman, 1991).

Just like I observed for *S. kuhlii*, the temporal activity of this unknown bat was affected by my light treatment. More specifically, foraging activity on treatment nights decreased in the first and fourth 10-minute intervals after sunset. This decreased foraging activity in the first 10 minute interval of lit nights might be a function of bat passes as it is accompanied by lower (mean number) of bat passes.

4.2.4 ***Saccolaimus saccolaimus***

Saccolaimus saccolaimus is common in Singapore and is another fast-flying species (Pottie et al., 2005). However when over water bodies and open fields, it has been observed to fly low to forage or drink (Murphy, 2001). My light treatment had no effect on it but the trend of its activity indices tends to be lower on treatment nights (especially its terminal buzzes and buzz ratio). *S. saccolaimus* in general had a low count of terminal buzzes and hence buzz ratio. This might be because *S. saccolaimus* is known to forage over a multitude of habitats such as the city, mangroves and forests etc. (Teo & Rajathurai, 1997; Pottie et al. 2005). It is possible Eco-lake is not its main foraging microhabitat, and instead, it just comes to drink. If that is so, the trend of its

activity indices might be explained by the fact that during treatment nights, *S. saccolaimus* is more reluctant to fly low over the water body to drink as it might be at greater vulnerability to predation which is already high during control nights.

4.2.5 *Myotis* spp.

As mentioned earlier, whichever species of *Myotis* I recorded, the chances are it is a slow-flying trawler, and therefore likely to be light adverse. In that case, I predicted that its activity is negatively affected by the light treatment. However, although my results were not significant, I actually observed the opposite trend where activity indices were higher on treatment nights for the nights *Myotis* spp. was observed. Also, ambient light was found to have a negative influence on activity. As mentioned in Section 4.1, this might be as *Myotis* bats are possibly more light sensitive and higher ambient light increases risk of predation (Scanlon & Petit, 2008).

Despite the negative effect of ambient light, most surprising was that on the nights *Myotis* spp. was present, the activity indices of *Myotis* spp. tended to be higher with the light treatment. Although again, I must stress, that this trend was not significant – a result that could be related to the large variance in my data. While this is in contrary to what I predicted, I speculate that it could be due to the fact that the *Myotis* bats that are able to commute to the pond are already those more tolerant to light. Bats in general show high levels of intraspecific variation (Barclay, 1999). Geggie & Fenton (1985) found that one species, *Eptesicus fuscus*, exhibited highly variable foraging activity at lamps with some never foraging at streetlights and others having higher activity than at dark areas. In some *Myotis* bats, intraspecific variation in their body mass and wing size have also been observed to affect their habitat use, with larger bats being more capable of faster flight and more likely to forage in open spaces (Kalcounis & Brigham, 1995; Jacobs, 1999). It could be possible that the *Myotis* bats that commute to the pond have

morphological characteristics that result in them being more light tolerant. If the LED light is attracting insects that *Myotis* bats prey on, this could explain why there is a higher buzz ratio (proxy of bat prey abundance) of for *Myotis* on treatment nights and not for the other species.

4.3 Limitations and future studies

Unlike the study by Russo et al. (2017) that found that artificial light caused a reduction in foraging and drinking activity, I did not observe such an effect. This could be as the study was done in Italy which has an entirely different climate to that of Singapore. In addition, Russo et al. (2017) conducted their survey at a cattle trough and illuminated the entire water surface. It could be possible that as I only illuminated a portion of the water surface of Eco-lake, there were other areas unlit that were still suitable for foraging and drinking. Bats in those areas may have then inadvertently passed by my study site. Nonetheless, the general trend of my data indicated that there was a negative effect of light for most of the observed bat species. The lack of significant results could be due to several factors which I believe can be explored in future studies.

For this study, I had a sampling period of 30 nights. However, in order to detect considerably rare bat species, it might be necessary to sample for periods greater than 45 nights (Skalak et al., 2012) which might also give more precise results (Krebs, 1989). It might also be informative to conduct this study at other water bodies in Singapore as depending on the location and size as there might be differing results. A water body located in the Central Catchment Nature Reserve might attract a different community of bats that will react differently to the presence of artificial light. As mentioned, the size of the water body might also play a factor, as smaller water bodies would have a greater proportion of water surface illuminated if the same light was used.

In addition, in Singapore most lights near water bodies in parks are kept on throughout the night. Therefore it might be worthwhile to increase the surveying time to determine if this influences bat activity throughout the night. However, a greater sampling effort also results in a vast amount of acoustic data to be analysed. While gathering acoustic data of bats is easy, the manual identification of bat passes to species can become a bottleneck (Stahlschmidt & Brühl, 2012). Multivariate identification software can be used to automatically identify calls to species (Parsons et al., 2000). However, this is not possible without a well established echolocation call library. The building an echolocation call library would therefore help faciliate future studies on bats in Singapore.

While it appears that there is no significant effect of light on bats at the current level of light intensity used in this study, only a single light was used. The presence of multiple lights along the shore of a water body illuminating the water surface might reduce the unlit area available for bats. In addition, testing further light intensities could determine the level of light threshold of bats which differs among species (Stone et al., 2012). The increase in the global demand for LED lighting and the fact that the Singapore government will be changing all its 95,000 HPSV streetlights to LED lights by 2022 (Abdullah, 2017) also calls for an urgent need to determine the ecological impacts of such a change.

5. CONCLUSION

This study aimed to determine the effect of an artificial light treatment on bats at an urban water body. The effect of the light had no significant effect on the overall activity of bats observed although for some species; light caused changes in their temporal foraging activity. By introducing a novel artificial light at an area of a water body that was previously dark, the results of my study can possibly be used as a baseline to inform future lighting decisions. The results of this study also opens the door to other possible future studies that can be done. Of high importance is the need to look at the ecological impacts of switching from current lights to LED lighting, which although are more energy efficient, might have detrimental effects on the bats in Singapore.

6. REFERENCES

Abdullah, Z. (2017). LTA installing smarter, energy-saving street lights. *The Straits Times*. Retrieved from http://www.straitstimes.com/singapore/lta-installing-smarter-energy-saving-street-lights

Acharya, L., & Fenton, M. B. (1999). Bat attacks and moth defensive behaviour around street lights. *Canadian Journal of Zoology*, 77(1), 27–33.

Arlettaz, R., Godat, S., & Meyer, H. (2000). Competition for food by expanding pipistrelle bat populations (*Pipistrellus pipistrellus*) might contribute to the decline of lesser horseshoe bats (*Rhinolophus hipposideros*). *Biological Conservation*, 93(1), 55–60.

Barclay, R. M. (1999). Bats are not birds—a cautionary note on using echolocation calls to identify bats: a comment. *Journal of Mammalogy*, 80(1), 290-296.

Bell, G. P. (1980). Habitat use and response to patches of prey by desert insectivorous bats. *Canadian Journal of Zoology*, 58(10), 1876-1883.

Blake, D., Hutson, A. M., Racey, P. A., Rydell, J., & Speakman, J. R. (1994). Use of lamplit roads by foraging bats in southern England. *Journal of Zoology*, 234(3), 453-462.

Boonman, A. M., et al (1998). Prey detection in trawling insectivorous bats: duckweed affects hunting behaviour in Daubenton's bat, *Myotis daubentonii*. *Behavioral Ecology and Sociobiology*, 44(2), 99–107.

Boyles, J. G., Cryan, P. M., McCracken, G. F., & Kunz, T. H. (2011). Economic importance of bats in agriculture. *Science*, 332(6025), 41–42.

Brook, B. W., Sodhi, N. S., & Ng, P. K. (2003). Catastrophic extinctions follow deforestation in Singapore. *Nature*, 424, 420–426.

Chan, K. W., Lim, K. K. P., & Leong, T. M. (2009). The Javan pipistrelle, Pipistrellus javanicus (Mammalia: Chiroptera: Vespertilionidae) in Singapore. *Nature in Singapore*, 2, 323–327.

Chin, S.C. (2008). *Biodiversity conservation in Singapore*. Retrieved from https://www.bgci.org/resources/article/0585/

Ciechanowski, M. (2002). Community structure and activity of bats (Chiroptera) over different water bodies. *Mammalian Biology*, 67(5), 276–285.

Cinzano, P., Falchi, F., & Elvidge, C. D. (2001). The first world atlas of the artificial night sky brightness. *Monthly Notices of the Royal Astronomical Society*, 328(3), 689–707.

Coleman, J. L., & Barclay, R. M. (2011). Influence of urbanization on demography of little brown bats (*Myotis lucifugus*) in the prairies of North America. *PLoS One*, 6(5), e20483.

Corben, C. (2009). *Bat Calls of Borneo*. Retrieved from http://users.lmi.net/corben/BatsOfBorneo.htm

Davies, T. W., Bennie, J., & Gaston, K. J. (2012). Street lighting changes the composition of invertebrate communities. *Biology Letters*, 8, 764–767.

Davies, T. W., Bennie, J., Inger, R., & Gaston, K. J. (2013). Artificial light alters natural regimes of night-time sky brightness. *Scientific reports*, 3(1722), 1–6.

Denzinger, A., & Schnitzler, H. (2013). Bat guilds, a concept to classify the highly diverse foraging and echolocation behaviors of microchiropteran bats. *Frontiers in physiology*, 4(164), 9-23.

Fabianek, F., Gagnon, D., & Delorme, M. (2011). Bat distribution and activity in Montréal Island green spaces: responses to multi-scale habitat effects in a densely urbanized area. *Ecoscience*, 18(1), 9–17.

Falchi, F., Cinzano, P., Elvidge, C. D., Keith, D. M., & Haim, A. (2011). Limiting the impact of light pollution on human health, environment and stellar visibility. *Journal of environmental management*, 92(10), 2714–2722.

Falchi, F., Cinzano, P., Duriscoe, D., Kyba, C. C., Elvidge, C. D., Baugh, K., ... & Furgoni, R. (2016). The new world atlas of artificial night sky brightness. *Science advances*, 2(6), e1600377.

Faure, P. A., Fullard, J. H., & Dawson, J. W. (1993). The gleaning attacks of the northern long-eared bat, Myotis septentrionalis, are relatively inaudible to moths. *Journal of Experimental Biology*, 178(1), 173-189.

Fukui, D. A. I., Murakami, M., Nakano, S., & Aoi, T. (2006). Effect of emergent aquatic insects on bat foraging in a riparian forest. *Journal of Animal Ecology*, 75(6), 1252–1258.

Gaston, K. J., & Bennie, J. (2014). Demographic effects of artificial nighttime lighting on animal populations. *Environmental Reviews*, 22(4), 323–330.

Gaston, K. J., Visser, M. E., & Hölker, F. (2015). The biological impacts of artificial light at night: the research challenge. *Philosophical Transactions of the Royal Society of London B: Biological Sciences*, 370.

Geggie, J. F., & Fenton, M. B. (1985). A comparison of foraging by *Eptesicus fuscus* (Chiroptera: Vespertilionidae) in urban and rural environments. *Canadian Journal of Zoology*, 63(2), 263-266.

Griffin, D. R., Webster, F. A., & Michael, C. R. (1960). The echolocation of flying insects by bats. *Animal behaviour*, 8(3), 141–154.

Grimm, N. B., Faeth, S. H., Golubiewski, N. E., Redman, C. L., Wu, J., Bai, X., & Briggs, J. M. (2008). Global change and the ecology of cities. S*cience*, 319(5864), 756–760.

Haeussler, U., & Erkert, H. (1978). Different direct effects of light intensity on the entrained activity rhythm in neotropical bats (Chiroptera, Phyllostomidae). *Behavioural processes*, 3(3), 223–239.

Haffner, M., & Stutz, H. P. (1985). Abundance of Pipistrellus *pipistrellus* and *Pipistrellus kuhlii* foraging at street-lamps. *Myotis*, *23*(24), 167-172.

Hale, J. D., Fairbrass, A. J., Matthews, T. J., Davies, G., & Sadler, J. P. (2015). The ecological impact of city lighting scenarios: exploring gap crossing thresholds for urban bats. *Global change biology*, 21(7), 2467–2478.

Hölker, F., Moss, T., Griefahn, B., Kloas, W., Voigt, C. C., Henckel, D., ... & Franke, S. (2010a). The dark side of light: a transdisciplinary research agenda for light pollution policy. *Ecology and Society*, 15(4), 13–23.

Hölker, F., Wolter, C., Perkin, E. K., & Tockner, K. (2010b). Light pollution as a biodiversity threat. *Trends in ecology & evolution*, 25(12), 681–682.

Hourigan, C. L., Catterall, C. P., Jones, D., & Rhodes, M. (2010). The diversity of insectivorous bat assemblages among habitats within a subtropical urban landscape. *Austral Ecology*, 35(8), 849–857.

Hughes, A. C., Satasook, C., Bates, P. J., Soisook, P., Sritongchuay, T., Jones, G., & Bumrungsri, S. (2011). Using echolocation calls to identify Thai bat species: Vespertilionidae, Emballonuridae, Nycteridae and Megadermatidae. *Acta Chiropterologica*, 13(2), 447–455.

Jacobs, D. S. (1999). Intraspecific variation in wingspan and echolocation call flexibility. *Acta Chiropterologica, 1(1),* 93-103.

Jones, G., Jacobs, D. S., Kunz, T. H., Willig, M. R., & Racey, P. A. (2009). Carpe noctem: the importance of bats as bioindicators. *Endangered species research*, 8(1-2), 93–115.

Jones, G., & Rayner, J. M. V. (1991). Flight performance, foraging tactics and echolocation in the trawling insectivorous bat *Myotis adversus* (Chiroptera: Vespertilionidae). *Journal of Zoology*, 225(3), 393–412.

Jones, G., & Rydell, J. (1994). Foraging strategy and predation risk as factors influencing emergence time in echolocating bats. *Philosophical Transactions of the Royal Society of London B: Biological Sciences*, 346(1318), 445–455.

Jung, K., & Kalko, E. K. (2010). Where forest meets urbanization: foraging plasticity of aerial insectivorous bats in an anthropogenically altered environment. *Journal of Mammalogy*, 91(1), 144–153.

Kalcounis, M. C., & Brigham, R. M. (1995). Intraspecific variation in wing loading affects habitat use by little brown bats (Myotis lucifugus). *Canadian Journal of Zoology*, 73(1), 89-95.

Kingston, T. (2010). Research priorities for bat conservation in Southeast Asia: a consensus approach. *Biodiversity and Conservation*, 19(2), 471–484.

Kingston, T., Jones, G., Akbar, Z., & Kunz, T. H. (2003). Alternation of echolocation calls in 5 species of aerial-feeding insectivorous bats from Malaysia. *Journal of Mammalogy*, 84(1), 205–215.

Korine, C., Adams, R., Russo, D., Fisher-Phelps, M. & Jacobs, D. (2016). Bats and water: anthropogenic alterations threaten global bat populations. InVoigt, C. C., & Kingston, T. (Eds.), *Bats in the Anthropocene: conservation of bats in a changing world* (pp 215–241). Springer.

Krebs, C. (1989) *Ecological Methodology*. 654pp. Harper & Row, New York.

Kuijper, D. P., Schut, J., van Dullemen, D., Toorman, H., Goossens, N., Ouwehand, J., & Limpens, H. J. G. A. (2008). Experimental evidence of light disturbance along the commuting routes of pond bats (Myotis dasycneme). *Lutra*, 51(1), 37.

Kunz, T. H. (1982). Roosting ecology of bats. In Kunz, T.H. (Ed.), *Ecology of bats* (pp 1–46). New York: Plenum Press.

Lane, D. J., Kingston, T., & Lee, B. P. H. (2006). Dramatic decline in bat species richness in Singapore, with implications for Southeast Asia. *Biological conservation*, 131(4), 584–593.

Leong, T. M., & Lim, K. K. P. (2009). Noteworthy microchiropteran records from the Bukit Timah and Central Catchment Nature Reserves, Singapore. *Nature in Singapore*, 2, 83–90.

Leong, T. M., Shunari, M., & Lim, K. K. (2010). The narrow-winged pipistrelle, *Pipistrellus stenopterus* (Dobson) in Singapore (Mammalia: Chiroptera: Vespertilionidae). *Nature in Singapore*, 3, 159–165.

Lim, K. P. & Leong, T.M. (2014). New record of the Horsfield's large-footed myotis in Singapore. *Singapore Biodiversity Records 2014*, 106–107.

Linley, G. D. (2016). The impact of artificial lighting on bats along native coastal vegetation. *Australian Mammalogy*.

Longcore, T., & Rich, C. (2004). Ecological light pollution. *Frontiers in Ecology and the Environment*, 2(4), 191–198.

Magurran, A. E. (2013). *Measuring biological diversity*. Blackwell Publishing.

McKenzie, N. L., Gunnell, A. C., Yani, M., & Williams, M. R. (1995). Correspondence Between Plight Morphology and Foraging Ecology in Some Palaeotropical Bats. *Australian Journal of Zoology*, 43(3), 241–257.

Mickleburgh, S. P., Hutson, A. M., & Racey, P. A. (2002). A review of the global conservation status of bats. *Oryx*, 36(1), 18–34.

Murphy, S. (2001). Observations of the'Critically Endangered' bare-rumped sheathtail bat *Saccolaimus saccolaimus* Temminck (Chiroptera: Emballonuridae) on Cape York Peninsula, Queensland. *Australian Mammalogy*, 23(2), 185-187.

National Parks Board (2014). *Singapore Botanic Gardens: Candidate World Heritage Site Nomination Dossier*. Retrieved from http://www.sbg.org.sg/images/UNESCO/UNESCO%20240114%20SBG%20Candidate%20WHS_Nom%20Doc_Final_2014-01%20opt.pdf

O'Farrell, M. J., & Gannon, W. L. (1999). A comparison of acoustic versus capture techniques for the inventory of bats. *Journal of Mammalogy*, *80*(1), 24-30.

Parsons, S., Boonman, A. M., & Obrist, M. K. (2000). Advantages and disadvantages of techniques for transforming and analyzing chiropteran echolocation calls. *Journal of Mammalogy*, 81(4), 927-938.

Perkin, E. K., Hölker, F., Richardson, J. S., Sadler, J. P., Wolter, C., & Tockner, K. (2011). The influence of artificial light on stream and riparian ecosystems: questions, challenges, and perspectives. *Ecosphere*, 2(11), 1–16.

Pottie, S. A., Lane, D. J., Kingston, T., & Lee, B. P. H. (2005). The microchiropteran bat fauna of Singapore. *Acta Chiropterologica*, *7*(2), 237–247.

Raghuram, H., Jain, M., & Balakrishnan, R. (2014). Species and acoustic diversity of bats in a palaeotropical wet evergreen forest in southern India. *Current Science,* 107(4), 631–641.

Ratcliffe, J. M., Elemans, C. P., Jakobsen, L., & Surlykke, A. (2013). How the bat got its buzz. *Biology Letters*, 9(2), 20121031.

Robert, C. (2016). Singapore has most light pollution in the world. *The New Paper*. Retrieved from: http://www.tnp.sg/news/singapore/singapore-has-most-light-pollution-world

Rowse, E. G., Harris, S., & Jones, G. (2016). The switch from low-pressure sodium to light emitting diodes does not affect bat activity at street lights. *PloS One*, 11(3), e0150884.

Rowse, E. G, Lewanzik, D., Stone, E.L., Harris, S., Jones, G. (2016b). Dark matters: the effects of artificial lighting on bats. *Bats in the Anthropocene: Conservation of Bats in a Changing World* (pp. 187-213).

Russ, J. (2012). *British bat calls: a guide to species identification*. Exeter: Pelagic publishing.

Russo, D., Cistrone, L., & Jones, G. (2012). Sensory ecology of water detection by bats: a field experiment. *PLoS One*, 7(10), e48144.

Russo, D., Ancillotto, L., Cistrone, L., & Korine, C. (2016). The Buzz of Drinking on the Wing in Echolocating Bats. *Ethology*, 122(3), 226–235.

Russo, D., Cistrone, L., Libralato, N., Korine, C., Jones, G., & Ancillotto, L. (2017). Adverse effects of artificial illumination on bat drinking activity. *Animal Conservation*

Rydell, J. (1991). Seasonal use of illuminated areas by foraging northern bats *Eptesicus nilssoni*. *Ecography*, *14*(3), 203-207.

Rydell, J. (1992). Exploitation of insects around streetlamps by bats in Sweden. *Functional Ecology*, 6, 744–750.

Rydell, J., Entwistle, A., & Racey, P. A. (1996). Timing of foraging flights of three species of bats in relation to insect activity and predation risk. *Oikos,* 76, 243–252.

Scanlon, A. T., & Petit, S. (2009). Effects of site, time, weather and light on urban bat activity and richness: considerations for survey effort. *Wildlife Research*, 35(8), 821-834.

Schober, W. (1997). *The bats of Europe & North America: knowing them, identifying them, protecting them*. Tfh Publications Incorporated.

Schoeman, M. C. (2015). Light pollution at stadiums favors urban exploiter bats. *Animal Conservation*, 19(2), 120–130.

Schubert, E. F., & Kim, J. K. (2005). Solid-state light sources getting smart. *Science*, 308(5726), 1274–1278.

Skalak, S. L., Sherwin, R. E., & Brigham, R. M. (2012). Sampling period, size and duration influence measures of bat species richness from acoustic surveys. *Methods in Ecology and Evolution*, 3(3), 490-502.

Stahlschmidt, P., & Brühl, C. A. (2012). Bats as bioindicators–the need of a standardized method for acoustic bat activity surveys. *Methods in Ecology and Evolution*, *3*(3), 503-508.

Stevens, R. D., & Willig, M. R. (2002). Geographical ecology at the community level: perspectives on the diversity of New World bats. *Ecology*, 83(2), 545–560.

Stone, E. L., Jones, G., & Harris, S. (2009). Street lighting disturbs commuting bats. *Current biology*, 19(13), 1123–1127.

Stone, E. L., Jones, G., & Harris, S. (2012). Conserving energy at a cost to biodiversity? Impacts of LED lighting on bats. *Global Change Biology*, 18(8), 2458–2465.

Stone, E. L., Harris, S., & Jones, G. (2015a). Impacts of artificial lighting on bats: a review of challenges and solutions. *Mammalian Biology-Zeitschrift für Säugetierkunde*, 80(3), 213–219.

Stone, E. L., Wakefield, A., Harris, S., & Jones, G. (2015b). The impacts of new street light technologies: experimentally testing the effects on bats of changing from low-pressure sodium to white metal halide. *Phil. Trans. R. Soc. B, 370*(1667).

Speakman, J. R. (1991). Why do insectivorous bats in Britain not fly in daylight more frequently?. *Functional Ecology*, 5, 518-524.

Thomas, D. W. (1988). The distribution of bats in different ages of Douglas-fir forests. *The Journal of Wildlife Management*, 52(4), 619–626.

Voigt, C.C., & Kingston, T. (2016). *Bats in the Anthropocene: Conservation of Bats in a Changing World* (pp. 2–3). Springer.

Voigt, C.C. & Lewanzik, D. (2011). Trapped in the darkness of the night: thermal and energetic constraints of daylight flight in bats. *Proceedings of the Royal Society, Series B*, 278, 2311–2317.

Wakefield, A., Broyles, M., Stone, E. L., Jones, G., & Harris, S. (2016). Experimentally comparing the attractiveness of domestic lights to insects: Do LEDs attract fewer insects than conventional light types?. *Ecology and Evolution*, 6(22), 8028-8036.

Wordley, C. F., Foui, E. K., Mudappa, D., Sankaran, M., & Altringham, J. D. (2014). Acoustic identification of bats in the southern Western Ghats, India. *Acta Chiropterologica*, 16(1), 213–222.

Zhu, G., Chmura, A., and Zhang, L. (2012). Morphology, echolocation calls and diet of Scotophilus kuhlii (Chiroptera: Vespertilionidae) on Hainan Island, south China. *Acta Chiropterologica*, 14(1), 175–181.

7. APPENDIX

Appendix A

Table 5: Echolocation call parameters used for species identification for species observed in this study.

Species	Location	Duration (ms)	Maximum frequency (kHz)	Minimum frequency (kHz)	Peak frequency (kHz)	Reference
Scotophilus kuhlii	Singapore	4.01 ± 0.03	84.90 ± 2.25	36.60 ± 0.46	43.30 ± 0.16	Pottie el al., 2005
	Lombok, Indonesia	2.30 – 3.20	51.20	40.60 − 41.20	41.60 − 42.20	McKenzie et al., 1995
	Hainan, China	2.90 – 8.10	64.20 – 103.20	31.00 – 44.10	41.90 – 51.40	Zhu et al., 2012
	India	2.20 – 3.50	52.40 – 61.30	42.10 – 44.90	44.00 – 47.00	Wordley et al., 2014
	India	3.20 ± 0.50	117.40 ± 9.20	41.00 ± 0.60	52.80 ± 4.60	Raghuram et al., 2014
Myotis adverus	Singapore	4.68 ± 0.10	82.50 ± 0.71	30.40 ± 0.36	46.20 ± 0.31	Pottie el al., 2005
	Australia	5.14 ± 0.84	80.18 ± 3.50	31.16 ± 2.99	46.49 ± 4.30	Jones & Rayner, 1991
Myotis horsfieldii	Thailand	3.17 ± 2.60	134.25 ± 9.60	38.38 ± 3.46	56.93 ± 7.98	Hughes et al., 2011
	India	1.54 – 6.70	50.75 –126.8	33.46 – 57.5	37.90 – 101.00	Wordley et al., 2014
Saccolaimus saccolaimus	Singapore	12.20 ± 0.08	23.50 ± 1.32	21.80 ± 1.42	22.60 ± 0.42	Pottie el al., 2005
	Thailand	3.85 ± 2.11	60.25 ± 12.09	17.75 ± 0.98	32.03 ± 8.85	Hughes et al., 2011
Pipistrellus stenopterus	Malaysia	13.80 ± 0.42	42.80 ± 2.01	28.00 ± 0.40	31.00 ± 0.49	Kingston et al., 2003

Appendix B

Table 6: AIC of each model type for the three bat activity indices (number of bat passes, total buzzes, buzz ratio) for all species and each species / species group. Poisson: GLM with Poisson distribution; Negative binomial: GLM with negative binomial distribution. Model with smallest AIC value (given in bold) was retained.

Species	Activity index	Poisson	Negative binomial
All bats	Bat passes	408.87	**318.06**
	Terminal buzzes	2100.3	**380.17**
	Buzz ratio	172.05	**70.31**
Scotophilus kuhlii	Bat passes	407.49	**307.57**
	Terminal buzzes	2400.3	**368.57**
	Buzz ratio	229.98	**76.23**
Unknown	Bat passes	263.37	**248.33**
	Terminal buzzes	447.70	**238.96**
	Buzz ratio	445.33	**45.238**
Saccolaimus saccolaimus	Bat passes	276.89	**222.01**
	Terminal buzzes	166.84	**130.44**
	Buzz ratio	145.09	**35.70**
Myotis spp.	Bat passes	845.77	**172.87**
	Terminal buzzes	1994.3	**171.68**
	Buzz ratio	624.65	**72.78**

Appendix C

Table 7: Effects of light treatment and environmental variables on the activity indices of each bat species. Table shows estimate values (±SE), the z-value F and the p-value for each species.

Species \ Activity index		Light	Temperature	Wind	Ambient light
**Overall species**	Bat pass	-0.032(±0.061)<br>F=-0.529<br>P=0.597	0.018(±0.021)<br>F=0.843<br>P=0.399	-0.018(±0.014)<br>F=-1.261<br>P=0.207	-0.706(±0.362)<br>F=-1.951<br>P=0.051
	Terminal buzz	-0.166(±0.263)<br>F=-0.629<br>P=0.529	0.076(±0.090)<br>F=0.843<br>P=0.399	0.040 (±0.062)<br>F=0.651<br>P=0.515	0.548(±1.567)<br>F=0.350<br>P=0.727
	Buzz ratio	-0.133 (±0.466)<br>F=-0.286<br>P=0.775	0.06(±0.162)<br>F=0.370<br>P=0.711	0.056 (±0.107)<br>F=0.526<br>P=0.599	1.381(±2.692)<br>F=0.513<br>P=0.608
S. kuhlii	Bat pass	-1.543e-02 (±7.893e-02)<br>F=-0.196<br>P=0.845	7.581e-03(±2.708e-02)<br>F=0.280<br>P=0.780	6.588e-03(±1.854e-02)<br>F=0.355<br>P=0.722	-1.214e-01(±4.705e-01)<br>F=-0.258<br>P=0.796
	Terminal buzz	-0.246(±0.314)<br>F=-0.782<br>P=0.434	0.095(±0.108)<br>F=0.880<br>P=0.379	0.057(±0.074)<br>F=0.778<br>P=0.436	1.456(±1.870)<br>F=0.779<br>P=0.436
	Buzz ratio	-0.144 (±0.442)<br>F=-0.326<br>P=0.744	0.103(±0.155)<br>F=0.665<br>P=0.506	0.061(±0.100)<br>F=0.612<br>P=0.541	1.861 (±2.512)<br>F=0.741<br>P=0.459
**Unknown**	Bat pass	-0.145(±0.093)<br>F=-1.559<br>P=0.119	0.030(±0.027)<br>F=1.114<br>P=0.265	-0.065(±0.019)<br>F=3.453<br>P=0.184	-1.627(±0.484)<br>F=-3.362<br>P=0.002
	Terminal buzz	-0.605(±0.329)<br>F=-1.838<br>P=0.066	0.022(±0.111)<br>F=0.198<br>P=0.843	-0.213(±0.077)<br>F=-2.762<br>P=0.220	-3.418(±1.954)<br>F=-1.749<br>P=0.080
	Buzz ratio	-0.310(±0.786)<br>F=-0.395<br>P=0.693	-0.015(±0.251)<br>F=-0.060<br>P=0.952	-0.123(±0.188)<br>F=-0.652<br>P=0.514	-0.956(±4.79)<br>F=-0.200<br>P=0.842

Saccolaimus saccolaimus	Bat pass	-0.146(±0.093)<br>F=-0.240<br>P=0.119	-0.017(±0.072)<br>F=-0.242<br>P=0.809	-0.059 (±0.050)<br>F=-1.186<br>P=0.236	0.682(±1.250)<br>F=0.547<br>P=0.5847
	Terminal buzz	-0.925(±0.554)<br>F=-1.609<br>P=0.095	-0.077(±0.176)<br>F=-0.437<br>P=0.662	-0.198(±0.132)<br>F=-1.502<br>P=0.133	1.543(±3.123)<br>F=0.494<br>P=0.621
	Buzz ratio	-0.582(±1.010)<br>F=-0.396<br>P=0.564	-0.070(±0.330)<br>F=-0.212<br>P=0.832	-0.116(±0.253)<br>F=-0.459<br>P=0.646	-0.274(±6.287)<br>F=-0.044<br>P=0.965
***Myotis* spp.**	Bat pass	-1.735(±1.046)<br>F=-1.658<br>P=0.097	-0.392(±0.360)<br>F=-1.088<br>P=0.277	0.054(±0.244)<br>F=0.221<br>P=0.825	-3.75(±4.229)<br>F=-2.291<br>P=0.022
	Terminal buzz	-1.736(±1.247)<br>F=1.392<br>P=0.164	-0.566(±0.435)<br>F=-1.299<br>P=0.194	0.207(±0.291)<br>F=0.710<br>P=0.478	-1.48(±0.328)<br>F=-2.303<br>P=0.021
	Buzz ratio	-0.095(±0.609)<br>F=-0.156<br>P=0.876	-0.152(±0.210)<br>F=-0.722<br>P=0.470	0.035(±0.150)<br>F=0.231<br>P=0.817	-2.26 (±4.01)<br>F=-0.953<br>P=0.341

MichelleGanWanJie_Thesis

ORIGINALITY REPORT

9%	6%	5%	2%
SIMILARITY INDEX	INTERNET SOURCES	PUBLICATIONS	STUDENT PAPERS

PRIMARY SOURCES

1	Submitted to National University of Singapore<br>Student Paper	2%
2	Emma Louise Stone. "Conserving energy at a cost to biodiversity? Impacts of LED lighting on bats", Global Change Biology, 03/2012<br>Publication	1%
3	Stone, Emma Louise, Stephen Harris, and Gareth Jones. "Impacts of artificial lighting on bats: a review of challenges and solutions", Mammalian Biology - Zeitschrift für Säugetierkunde, 2015.<br>Publication	1%
4	Bats in the Anthropocene Conservation of Bats in a Changing World, 2016.<br>Publication	<1%
5	edepot.wur.nl<br>Internet Source	<1%
6	journals.plos.org<br>Internet Source	<1%
7	Cucurachi, S., R. Heijungs, W.J.G.M.	<1%

Peijnenburg, J.F.B. Bolte, and G.R. de Snoo. "A framework for deciding on the inclusion of emerging impacts in life cycle impact assessment", Journal of Cleaner Production, 2014.
Publication

---

8 etheses.whiterose.ac.uk
Internet Source
<1%

---

9 Lacoeuilhe, Aurelie, Nathalie Machon, Jean-François Julien, Agathe Le Bocq, and Christian Kerbiriou. "The Influence of Low Intensities of Light Pollution on Bat Communities in a Semi-Natural Context", PLoS ONE, 2014.
Publication
<1%

---

10 eprints.eriub.org
Internet Source
<1%

---

11 Maria D. Adams. "Effect of lights on activity levels of forest bats: increasing the efficiency of surveys and species identification", Wildlife Research, 2005
Publication
<1%

---

12 www.conservationevidence.com
Internet Source
<1%

---

13 de Jong, M., J. Q. Ouyang, A. Da Silva, R. H. A. van Grunsven, B. Kempenaers, M. E. Visser, and K. Spoelstra. "Effects of nocturnal illumination on life-history decisions and
<1%

fitness in two wild songbird species", Philosophical Transactions of The Royal Society B Biological Sciences, 2015.
Publication

14	ageconsearch.umn.edu<br>Internet Source	<1%
15	Smart, R.. "Lizards and landscapes: integrating field surveys and interviews to assess the impact of human disturbance on lizard assemblages and selected reptiles in a savanna in South Africa", Biological Conservation, 200503<br>Publication	<1%
16	Submitted to University of Leeds<br>Student Paper	<1%
17	Submitted to Napier University<br>Student Paper	<1%
18	Submitted to University of Leicester<br>Student Paper	<1%
19	etheses.bham.ac.uk<br>Internet Source	<1%
20	Narendra, Ajay, Samuel F. Reid, and Chloé A. Raderschall. "Navigational Efficiency of Nocturnal Myrmecia Ants Suffers at Low Light Levels", PLoS ONE, 2013.<br>Publication	<1%
21	docs.serdp-estcp.org<br>Internet Source	<1%

22	people.ucsc.edu<br>Internet Source	<1%
23	www.bats.org.uk<br>Internet Source	<1%
24	dspace.stir.ac.uk<br>Internet Source	<1%
25	www.plan-actions-chiropteres.fr<br>Internet Source	<1%
26	commons.lib.jmu.edu<br>Internet Source	<1%
27	Broders, Hugh G., C. Scott Findlay, and Ligang Zheng. "EFFECTS OF CLUTTER ON ECHOLOCATION CALL STRUCTURE OF MYOTIS SEPTENTRIONALIS AND M. LUCIFUGUS", Journal of Mammalogy, 2004.<br>Publication	<1%
28	fundamentaljournals.org<br>Internet Source	<1%
29	zidapps.boku.ac.at<br>Internet Source	<1%
30	Michael A. Menzel. "Effects of group selection silviculture in bottomland hardwoods on the spatial activity patterns of bats", Forest Ecology and Management, 20020615<br>Publication	<1%

31	Line Kessel. "Corneal fluorescence in relation to genetic and environmental factors: a twin study", Acta Ophthalmologica Scandinavica, 10/2003<br>Publication	<1%
32	www.intechopen.com<br>Internet Source	<1%
33	en.wikipedia.org<br>Internet Source	<1%
34	www.wwct.org.uk<br>Internet Source	<1%
35	www.eurobats.org<br>Internet Source	<1%
36	www.darkskyvic.org<br>Internet Source	<1%
37	www.batcon.org<br>Internet Source	<1%
38	www.ajcb.in<br>Internet Source	<1%
39	www.ci.benicia.ca.us<br>Internet Source	<1%
40	www.altwym.nl<br>Internet Source	<1%
41	www.fs.fed.us<br>Internet Source	<1%

42	www.kentbap.org.uk<br>Internet Source	<1%
43	web.unitn.it<br>Internet Source	<1%
44	www.rafbat.org<br>Internet Source	<1%
45	Perkin, Elizabeth K., Franz Hölker, and Klement Tockner. "The effects of artificial lighting on adult aquatic and terrestrial insects", Freshwater Biology, 2014.<br>Publication	<1%
46	jeb.biologists.org<br>Internet Source	<1%
47	www.nature.nps.gov<br>Internet Source	<1%

EXCLUDE QUOTES OFF

EXCLUDE BIBLIOGRAPHY ON

EXCLUDE MATCHES OFF
