# Supplementary material for: Ecological impacts of the LED-streetlight retrofit on insectivorous bats in Singapore
Source: PLoS One. 2021 May 26;16(5):e0247900. doi: 10.1371/journal.pone.0247900 (PMC8153503; doi:10.1371/journal.pone.0247900)
Supplement: S6 Appendix — (PDF) [file pone.0247900.s006.pdf]

The switch from HPS to LED streetlamps – impacts on insects

YAO XINYI

A0131172W

A thesis submitted to the
Department of Biological Sciences
National University of Singapore
in partial fulfilment for the
Degree of Bachelor of Science with Honours
in
Life Sciences

Life Sciences Honours Cohort
AY2017/2018 S1

DECLARATION

I hereby declare that this thesis is my original work and it has been written by me in its entirety. I have duly acknowledged all the sources of information which have been used in the thesis.

This thesis has also not been submitted for any degree in any university previously.

Yao Xinyi

____

Name of Student

09 April 2018

Acknowledgements

Final year project was one of the toughest challenges I have faced to date, and never have I regretted my decision to embark on this journey. This project would not have been possible without the people around me, who have given me ample encouragement and support for the entire year.

I would like to thank Dr Joanna Coleman. Thank you for taking me as your FYP student despite not knowing me at all. I will always remember how we had a discussion regarding "Conservation" in your office when we first met, your straightforwardness was so impressionable and cool! This project would be more arduous without your constant guidance and encouragement. Your passion for ecology is truly inspirational!

I would like to express my gratitude to Professor John Carson Allen Jr, from Duke-NUS Medical School, for helping me to run some of the GLMM modelling and allowing me to understand different types of statistical analysis better.

Also, many thanks to Singapore Land Transport Authority (LTA) for permitting me to mount insect traps on their lamp post. I would especially like to thank Mr Ethan Koh for LTA for setting time aside to meet me and answer all my queries.

My parents, who accompanied me to the field, staring at streetlamps and getting weird looks from the members of the public with me. Thank you for being tolerant of my evening absences and mood swings.

A huge thank you to my fellow FYP mates, Angela, Cheryl, Roanna and Kenneth for tolerating my nonsense and giving me constructive feedbacks during our Spinelli meetings.

Last but not the least, a callout to my friends, thank you for being by my side this year. To Kaijie, I would never forget the days where we pushed a ladder from point to point and got mistaken as construction workers. A big thank you for listening to all my rants and chatters about FYP and suffering from a headache and potential myocardial infarction when you edited this thesis!

Table of Contents

Acknowledgement .... **i**

Tables of Contents .... **ii**

Abstract .... **iii**

Introduction **1**

Material and Methods **7**

Site selection 7

Insect Trap Setup 8

Identification of Insects 8

Microhabitat Variables 8

Weather information 10

Landscape Variables 10

Distance to Water, Green Area 10

Quantifying Urbanization 11

Data Analysis 12

Results **14**

Richness 15

Evenness 16

Diversity 17

Abundance 18

Biomass 19

Discussion **20**

Insect Richness, Abundance and Light Type 20

Abundance and Rainfall 21

Biomass of Insect and Light Type 22

Overall Levels of Light Pollution 24

Implications 25

Future work **26**

Conclusion **28**

References **29**

Abstract

With most nations striving to meet their targets for the reduction of greenhouse gas emissions, more authorities are switching to more energy- and cost-efficient, lighting strategies. Singapore aims to complete its island-wide replacement of high-pressure sodium (HPS) with light-emitting diode (LED) lamps by 2022. However, these retrofits have proceeded with very few studies of the ecological impacts of LED lights. Most importantly, there have not been any studies conducted in the Tropics or jurisdictions with severe light pollution, as in Singapore. I carried out a before-after control impact experiment to address this knowledge gap as part of a twinned study that investigates the effect of Singapore LED retrofit on insect and their bat predators in Singapore. Using sticky traps, I sampled insects, identified them and estimated insect abundance, biomass and diversity. Traps at LED lights captured fewer families and fewer individuals, but insect biomass did not differ significantly between the two light types. Thus, an unintended consequence of LED retrofit might be a reduction of insect mortality near artificial lights due to reduced attraction, which has implications for predators of phototactic insects that use lamps as hunting grounds.

Introduction

Artificial light at night (ALAN) is arguably one of the most distinguishing features of urban areas, representing a major alteration to nocturnal landscapes[1] with corresponding effects on organisms and ecosystems. These far-reaching consequences have become the focus of many ecological studies, with various well-documented negative impacts of ALAN on organisms[2],such as the disorientation observed in the hatchling turtles[1]. ALAN is known to influence many behavioural attributes, and to potentially affect predator-prey relationships, disrupt circadian rhythms and alter insect species distribution, among other effects[2].

Despite these undesirable consequences, artificial lights continue to be used extensively, with global demand increasing at about 6% per year and contributing more than 19% to total global electricity consumption[3], which is a major contributor to global climate change[3]. Consequently, with nations striving to meet their commitments under the UNFCCC, jurisdictions are placing more emphasis on developing new lighting technologies that have lower greenhouse gas emissions[4]. One such technology is light emitting diodes (LEDs), which are increasingly being installed to replace older technologies, such as low-pressure (LPS) and high-pressure sodium (HPS) vapour lights[2,4]. LEDs have several advantages over sodium-vapour lights, such as reduced energy consumption[5], greater design flexibility and light quality, longer lifespans and lower maintenance costs[4,6].

In Singapore, the dominant outdoor lighting technology is HPS lamps. Differentiating them from LED lights is simple. HPS lights have a lower colour temperature and emit an orangish light, whereas LEDs consist of a blue LED bulb coated with phosphorus to give off a full spectrum white light[2]. LED lights have a higher colour rendering index

(meaning, objects appear more accurate to colour), and ensures greater visibility of targets, regardless of its colour of the target. Indeed, this visibility advantage, i.e., improved road safety, is one further reason why many authorities are implementing LED lighting[7].

However, this has far-reaching implications for phototactic insects. Although insects represent 57% of animal species, the modern extinction of insects is largely overlooked[8]. By conservative estimates, a million species of animals are predicted to go extinct by 2050, with 57,000 of these being insects[8]. Quoting Dunn[8], “the biodiversity crisis is undeniably an insect biodiversity crisis”. Insects play a crucial role in ecosystem processes[9], providing critical ecosystem services, such as pollination, seed dispersion and decomposition[10]. The loss of insect species would, therefore, be expected to disrupt of such services. A well-publicized example these days is the adversely affected dispersal rates of plants if their pollinators, such as *Apis mellifera* (honeybees), disappear. The removal of decomposers, such as blowflies, could result in the accumulation of organic waste matter[11]. Also, as a crucial linkage in the food chain, loss of a particular species of insects could alter interactions among remaining species[12]. Despite all this, the value of insects is still grossly underappreciated, and their conservation thus relatively neglected[8].

Both LED, and HPS lights emit wavelengths that fall within the spectrum of maximum sensitivity of the photoreceptor response by bichromatic and trichromatic insects[13]. Many insects, particularly nocturnal ones, exhibit positive phototaxis. Several hypotheses attempt to explain the underlying mechanism. One relates visibility or wavelength to the insect’s attraction to light. All insects express rhodopsin with absorption peaks in the UV range, resulting in a higher sensitivity towards shorter light wavelength regions of the light spectrum[14]. Therefore, UV content of lamps would likely be a strong determining factor in determining insect attraction to artificial lights[15].

Alternatively, Scherer & Kolb (1987) suggested that insects possess photoreceptors that more than just for colour vision. Once activated by a specific wavelength of light, these photoreceptors can trigger wavelength-selective behaviours. For instance, moths in the genus *Pieris* exhibit different responses corresponding to the maximal absorbance at different wavelengths, namely escape at 370nm, feeding at 450nm and 600nm, drumming at 560nm and egg-laying at 540nm[16]. Therefore, the common observation of moths hovering around street lamp could be a wavelength-specific behaviour.

Finally, artificial lights may cause navigation errors[17]. Nocturnal insects typically use celestial objects to navigate. However, it is possible that insects mistakenly perceive artificial light sources as celestial bodies, in which case, they initiate equiangular spiral path towards the light source[15].

Regardless of the mechanism, ALAN has proven to have detrimental effects on insect populations, mainly via increased mortality. Einsenbeis and Hänel (2009)[18] estimated that approximately 360 million insects are killed each year by streetlamps in a German town with only 20,000 streetlamps. By extrapolation, over 3.42 billion insects are killed at streetlamps every year in Singapore.

Streetlights contribute to insect mortality in various ways. Many are killed by direct contact with hot light surfaces (as in the German town mentioned above)[19]. Others become disoriented and fly around the light continuously, ultimately dying of exhaustion[15]. Another factor to consider is greater predation risk where lighting can make insects more visible, to the extent that some predators have adapted to use these lights as hunting grounds[20].

Another cause of insect mortality is attributable to the absorption of specific wavelength of light by particular chromophores, or photosensitizers that some insects possess in their tissues. This absorption could generate free radicals that provoke fatal tissue damage[21].

ALAN also has sublethal effects on insects, e.g., by disrupting their circadian rhythms, basal activities and even reproductive success[18]. Again, given their crucial ecological roles, high mortality could have broad ecosystem effects, as well as economic ones, e.g., on agriculture. Also, illuminated spaces under artificial lights could become areas where diseases vectors come into contact with humans, facilitating the spread of infectious disease such as Chagas, leishmaniasis and malaria[22].

With an estimated 31% growth in market shares in 2010[23] , the global LED market is predicted to have a net worth of S$105 billion by 2020, representing close to 60% of the total lighting market[24]. It appears that we are looking at the future world in which LED lights are the norm when it comes to outdoor lighting technology, and so investigating the potential effects of these retrofits on biodiversity is timely.

Although LED retrofits are clearly a step in the right direction when it comes to climate change mitigation, these global retrofits are proceeding with little to no knowledge of the ecological impacts of LEDs, mainly because, very few studies have been conducted.

Studies of the attractiveness of LED lights to insects have yielded mixed results. One showed that insects were less attracted to LEDs than to HPS- or other light types[13], while another showed that all colour temperatures of LED lights were more attractive to insects than to LPS lights[25]. Similarly, insects exhibit greater positive phototaxis toward LED- than toward HPS lights in yet another study[26]. It is worth noting that many studies (e.g., 17, 18) have observed greater insect attraction to white lights (though not LEDs) than to other lights[20,27].

However, all the studies mentioned above were conducted in the temperate zone, and in relatively dark locations. Results from similar studies conducted in temperate climates [13,25,26] also might not apply to Singapore's context due to differences in life history [28], predator-prey relationships, adaptations of the insect, caused by environmental variability[29]. Furthermore, both studies[19,25] only sampled a single location, and there could be significant variation between sites. Consequently, there is no information about the possible ecological impacts of LED lights in the Tropics or locales with severe light pollution, such as Singapore[30].

Singapore is unique among tropical nations for having the highest rate of urbanisation (100 % of its population urbanised). Singapore's urbanisation has also occurred very rapidly, and massive deforestation and subsequent urbanisation have reduced its primary forest cover to just 0.2% of what it was initially. Yet, many native species survive despite the widespread habitat destruction[31].

Singapore is also one of the most light-polluted cities in the world[30], and, like many countries, it is pursuing energy efficiency via an island-wide streetlight retrofit. By 2022, the Land Transport Authority (LTA) aims to have all 95,000 street lights converted to LEDs[32]. The new lights will incorporate cut-off features, ensuring that lights are projected horizontally and downwards, thereby minimising light wastage[32]. Although the intent to reduce environmental impacts is evident, the ecological effects of LED lighting remain largely unknown.

I sought to investigate the comparative attractiveness of HPS and LED lights to insects, as one half of a twinned study on the ecological impacts of the retrofit (the other half investigates the impact on bats). This study is the first study that addresses this knowledge gap.

Since many studies have observed more insects being attracted to white light as compared to other types of lights (e.g. 26, 31, 33), I hypothesized that insect positive phototaxis is influenced by the colour temperature of the light, and I expected more insects to be attracted to the white light emitted by the LED light as opposed to the orange-hued illumination from HPS lights.

Research findings have also shown that light attraction might be determined by sensitivity to light, which is in turn related to body size[33]. It is assumed that large insect species have larger eyes, which confers them higher sensitivity as compared to smaller insect species with correspondingly smaller eyes[34]. Therefore, I expected estimated biomass of the insects found at LED lights to be higher than those insects found at HPS lights.

After considering the available literature, I proceeded to carry out my experiment with the hypothesis that LED lights would attract larger insects, and in greater numbers as compared to HPS lights, with my methodology detailed below.

Material and Methods

1. Site Selection

My study sites were on the mainland of Singapore, an urbanised city-state south of the Malay Peninsula. Although it has a tropical climate with no distinct seasonality, it experiences a wetter monsoon season from November to January. With the permission from LTA, I conducted a before-after control impact design experiment to study the effect of LED and HPS streetlights on insects. I obtained a list from LTA indicating where there were streets with retrofits completed near streets with HPS lamps, I then selected five study sites according to the following criteria: similar lamp height, power output, colour temperature and illuminance.

These five replicate streets each consist of a paired treatment street (illuminated by LED; blue pins, Fig. 1) and control street (illuminated HPS; yellow pins, Fig. 1) lamps. Control and treatment streets were separated by at least 400 m ($\bar{x}$ = 650 ± 380 m), and sites were at least 5km apart ($\bar{x}$ = 7.43 ± 5.28 km).

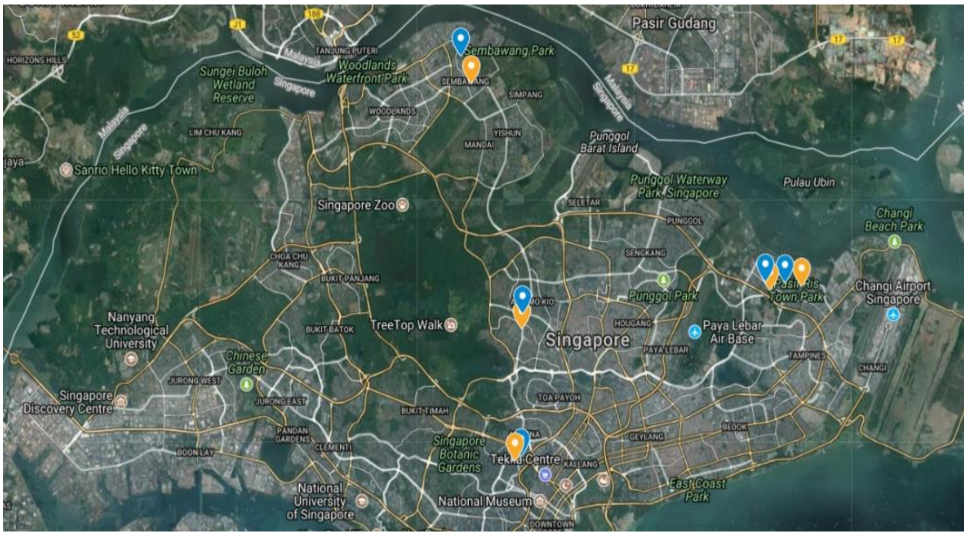

Figure 1. Location of the replicate sites.

Google Map showing the location of the five replicate paired sites, yellow pin indicates street illuminated by HPS lights, while blue indicates LED lights.

2. Insect trap setup

I sampled insects from October 2017 through the beginning of February. I only sampled insects on nights it did not rain. I sampled one site per night, two to three nights per week, rotating among sites. Ultimately, I conducted 25 field observations and sampled each site five times during the study period.

On each sampling night, I mounted sticky traps on randomly selected HPS and LED lamp posts. I wrapped a plastic sheet precoated with Tanglefoot onto a section of PVC pipe (30-cm long, 10-cm in diameter). Tanglefoot is a biodegradable and non-toxic adhesive, sticky enough to capture all but the largest, strongest insects, but not sticky enough to capture bats (J. Coleman, pers. comm.). I secured the sticky traps to the lamp posts using an Evenk knot at 7 pm and removed them at the end of the two-hour observation period.

3. Identification of Insects

In the laboratory the following day, I removed the trapped insects and observed them under a dissecting microscope, which allowed me to identify each one to the family level by using dichotomous keys[35] and other references (e.g., 37, 38, 39)[36, 37, 38]. Using a stage micrometre with 0.1mm interval, I measured the length of each specimen (undamaged ones only) from the head (excluding the antennae) to the tip of the abdomen (excluding cerci), to the nearest 0.1mm. This allowed me to estimate the biomass of each insect with order- and family-specific allometric formulae[39–43]. Measuring the dry weight was neither possible (because removing the Tanglefoot is not possible), nor ecologically meaningful (because the wet weight is what matters to bats). I could then calculate average biomass per individual in each trap

Microhabitat Variables

I recorded abiotic environmental variables to control for their effects.

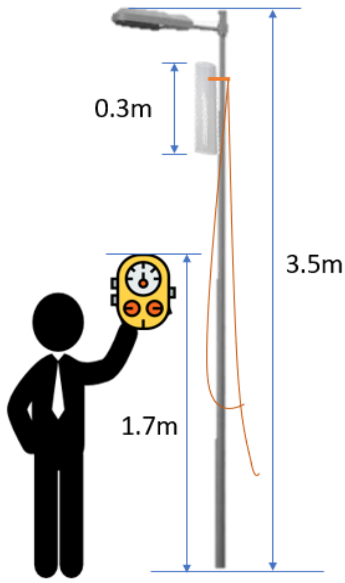

Figure 2: Measuring light intensity.

Diagram showing the setup of the insect trap and measurement of light intensity.

I measured light intensity with a Digital 50000 Lux Meter LX1010B Digital Light Level Photo Light Sensor, which I held directly under the lamp at the height of 1.7m (Fig. 2). I took these measurements at the end of the observation period to minimise the effects of ambient sunlight at dusk on the reading.

1) Weather info from NEA

I obtained weather data, specifically total daily rainfall, mean temperature and mean wind speed from the NEA Daily Historical Records[44].

Landscape Variables

1) Measuring the distance to water source, green area

I measured the distance to the nearest water source using the distance tool on Google Maps 9.73.3[45]. I defined water sources as water bodies including rivers, canals, ponds, swimming pools and reservoirs. This variable is ecologically meaningful because the distributions of certain invertebrates, such as midges (Chironomidae), which depend on water bodies for part of their life cycles, are linked to those of water bodies[46]. I also measured the distance to the nearest green space in a similar manner. Green spaces include grasslands, nature reserves, patches of forest and managed parks that are at least $0.05km^2$ in area.

2) Quantifying Urbanisation

Increasing urbanisation has a proven negative impact on insect persistence[47], making it imperative to assess the degree of urbanisation in the vicinity as well. I quantified urbanisation by the green cover. I plotted the coordinates of each lamp post in Google Maps 9.73.3 [45], and then overlaid a 20 by 20 grid over the 1km x 1km quadrant around it (with the post at the centre). I assigned an arbitrary value of 1 to grid squares with more than half their area as green cover (Fig. 3a, 3b).

$$Urbanization\ Index = 1 - \left(\frac{\sum_{1}^{200} Grids\ with\ "1"}{20 \times 20} \right)$$

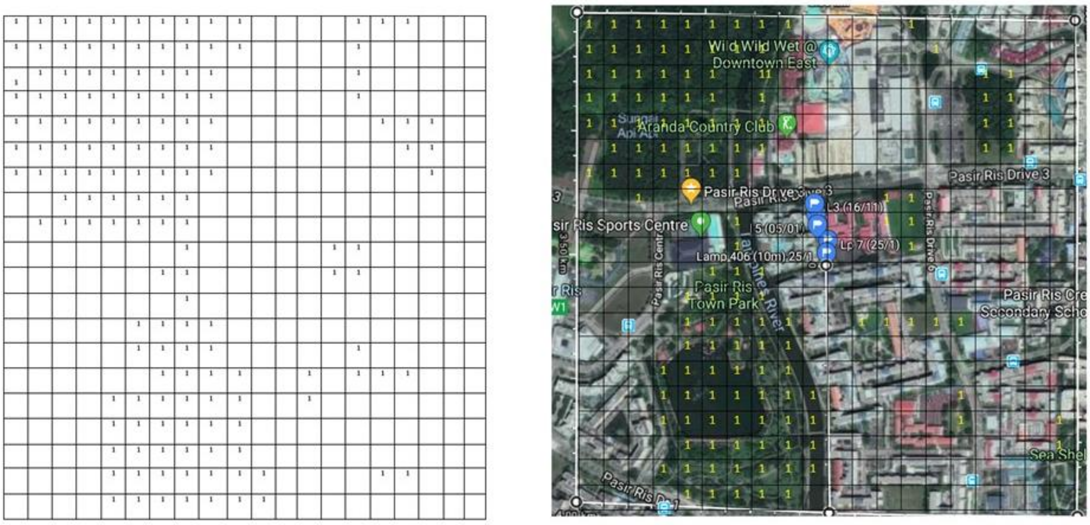

Figure 3: Quantifying Urbanization of the surrounding landscape.

Figure 3a (left): 20 by 20 grid overlaid onto the 1km by 1km quadrant. I assigned "1" to the grids if the green area takes up 50% of the area (in yellow). Figure 3b (right): 160 grids with value 1. Hence, urbanization index = 1-(160/400)

Data Analysis

I calculated five measures of insect assemblage parameters in each light type for every trap. The first is family richness or the number of insect families captured per trap. The second is the Berger-Parker index, one of the measures of dominance. The third was the Shannon-Wiener index, which considers both family richness and evenness when determining whether diversity differs among different light types. The fourth and the fifth were the abundance or the number of insects captured for individual light trap and the average biomass of an insect captured in each light trap. I used generalized linear mixed models (GLMM) with a normal distribution in Statistical Analysis System University Edition[48] to analyse my data.

The following predictors were included in my GLMM model: Light type as the fixed variable, site as a random variable and covariates encompass urbanisation, distance to water source, distance to green area, rainfall and light intensity while response variable includes, richness, diversity, evenness, biomass and abundance of insects. I intended to add mean daily temperature and average wind speed into the model, but the data points available from the NEA website proved to be insufficient. Furthermore, there are minimum temperature and wind variation in the tropics. Thus, these variables were excluded.

Using a two-tailed test and a rejection criterion of α=0.05 for the regression analysis of various dependent variables, I conducted a GLMM with repeated measures starting with a fully saturated model, eliminating non-significant terms sequentially until a reduced model with only the statistically significant terms were left. I tested for the normality of the residuals (Shapiro-Wilk test, normal quantile plot), checked for multicollinearity (PROC REG, SAS), homogeneity of variance (spread versus level plot, Levine test) and

absence of undue influence (no correlation between residuals and covariate) to ensure that the assumptions of the model were met. Then, I assessed the model fit using Akaike's Information Criterion (AIC), with a smaller value indicative of a better fit. I also had to evaluate the validity of using repeated measure analysis as I captured insects five times at each site. I tested for the effect of site on the different response variables with random effects model (restricted maximum likelihood model estimation) in SAS. As it turned out, the site did not have significant effects on the response variables, and thus it was removed from all analyses.

Since the proportion of orders and families of insects captured were normally distributed, I compared the proportion of four most abundant orders and families of insects found at each light type by using a series of Chi-Squared tests. I decomposed the light type*insect into four two-way tables comparing the proportion of a single insect against all the others combined. All statistical and descriptive analyses were performed using SAS.

Results

In all, I collected 523 insects, from 16 different orders on 25 nights of sampling, and identified all of them. I captured an identical number of orders for both HPS and LED lights. HPS lamp sites yielded 350 individuals belonging to 59 different families while 1 collected 173 insects from 34 families from the LED light traps (Table 1).

The most commonly captured insects belonged to the orders Hymenoptera, Hemiptera, Diptera and Coleoptera; consisting of more than 95% of all the insects captured. A greater proportion of Dipteran (χ^2_1= 23.35, P = 0.00) and Hemipteran (χ^2_1 = 8.76, P = 0,00) was observed for HPS and LED lights respectively, while the proportion of the Coleopterans and Hymenopterans did not differ among light types (Coleopterans: χ^2_1 = 0.76, P > 0.05, Hymenopterans: χ^2_1 = 3.79, P > 0.05).

Table 1: Orders and the number of individuals found at each light type.

The richness and the abundance of the insects captured at LED lights is approximately half of that found at HPS lights.

LED			HPS		
**Orders found**	**No. of families present**	**No. of Individuals**	**Orders found**	**No. of families present**	**No. of Individuals**
Hymenoptera	2	76	Diptera	16	136
Hemiptera	9	34	Hymenoptera	9	123
Diptera	10	31	Coleoptera	12	43
Coleoptera	8	26	Hemiptera	2	36
Ephemeroptera	1	2	Lepidoptera	4	5
Trichoptera	2	2	Psocoptera	1	3
Arachnida	1	1	Ephemeroptera	14	2
Orthoptera	1	1	Megaloptera	1	2
Total	34	173		59	350

Richness

Family richness differs between the two light types ($F_{1,9}$ =18.05, P = 0.00). Traps at HPS lights attracted insects from 59 families, whereas traps at LED lights captured 34 families. Insects from the Formicidae family were most abundant, accounting for 43% and 33% of insects at HPS and LED traps, respectively (Fig. 4). Comparing the proportion of the four most abundant families (Figure 4),, leafhoppers and ants were found in greater proportion at LED lights. (Cicadellidae (leafhoppers): χ^2_1 = 9.81, P =0.00, Formicidae (ants): χ^2_1 = 9.93, P=0.00, Chironomidae (non-biting midges): χ^2_1 = 0.72, P > 0.05, Scarabaeidae (scarab beetles): χ^2_1 = 0.96, P > 0.05).

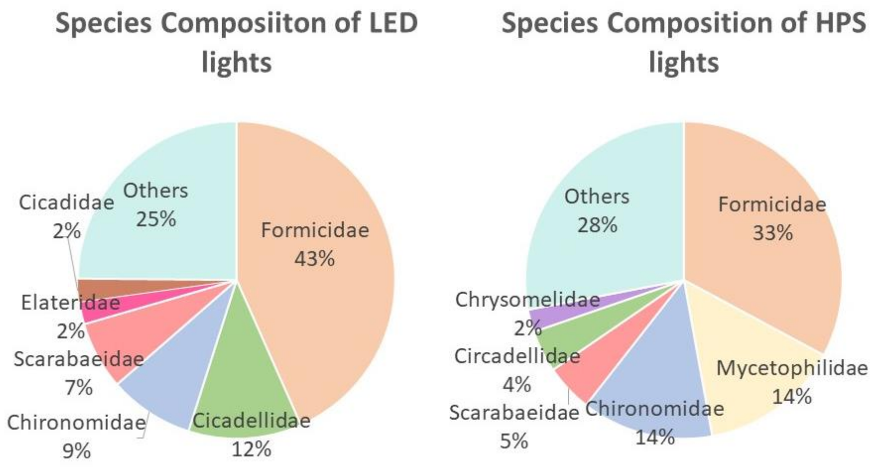

Figure 4: Pie chart showing the family composition found at each light type.

Only the top 6 most abundant families are shown, the remaining families are categorized as "Others". The "others" of LED lights consist of 28 families while "others" in HPS lights consist of 53 families.

Evenness

Rank abundance curve of LED and HPS is shown in blue and orange respectively (Fig.5). Family evenness at LED seems to be slightly lower at LED lamps, as represented by the less steep slope observed in the rank abundance curve (Fig. 5). The slope of the rank abundance curve indicates evenness. Greater evenness is found in the community if the slope tends to 0.

To confirm if there is a difference between the evenness of the assemblage between HPS and LED lights, I modelled Berger-Parker index of each trap as the response variable; the reduced GLMM model showed that insect assemblage dominance does not differ between light types. Hence, evenness found in both light types do not differ from each other ($F_{1,9}$=1.72, P = 0.22).

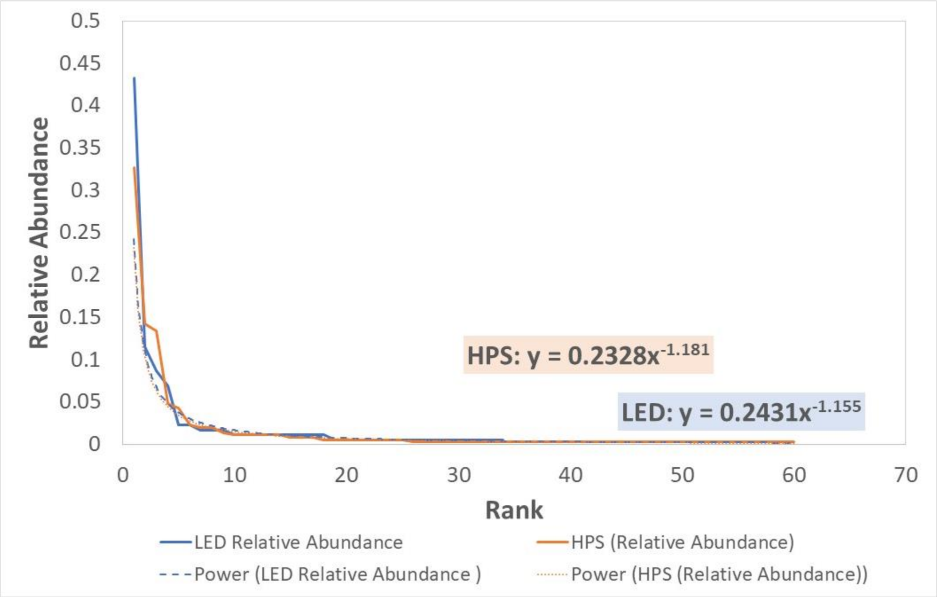

Figure 5: Rank abundance curve of LED and HPS lights.

LED has a lower family richness than HPS. However, the evenness found in LED lights is not different to that of HPS. ($F_{1,9}$=1.72, P = 0.22)

Diversity

I conducted statistical analysis of Shannon index (Fig. 6) using GLMM, which shows that the diversity between HPS and LED lights were not different ($F_{1,9}$ = 3.73, P=0.09).

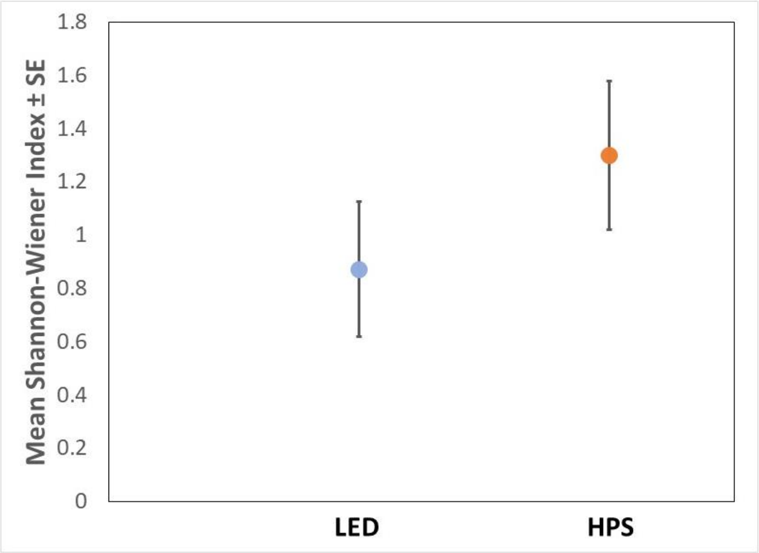

Figure 6: Mean Shannon-Wiener index of LED and HPS.

Statistical analysis shows that diversity in HPS and LED do not differ from each other ($F_{1,9}$ =3.73, P=0.09).

Abundance

Modelling the number of insects captured per trap revealed that HPS lights yielded a greater abundance of insects as compared to LED lights ($F_{1,9}$=6.12, P=0.04) (Fig. 7). Also, average rainfall shows a positive association with the number of insects caught under the streetlamps (R^2=0.07, Adjusted R^2=0.05, $F_{1,38}$=4.37, P=0.04).

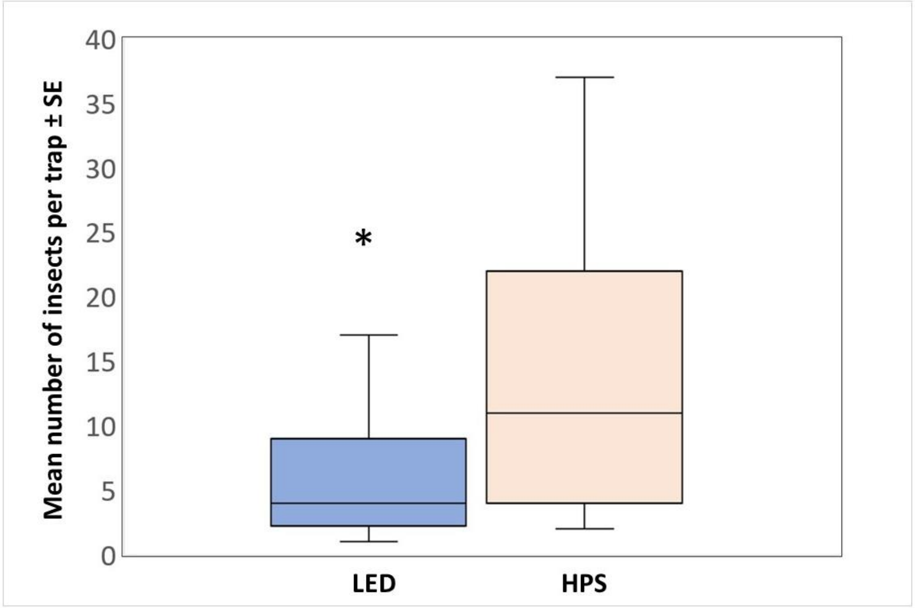

Figure 7: Number of the insect caught in the trap of each light type.

More insects were caught in the traps of the HPS lights as compared to the LED lights. The asterisk (*) shows that light type influences abundance ($F_{1,9}$=13.78, P=0.01).

Biomass

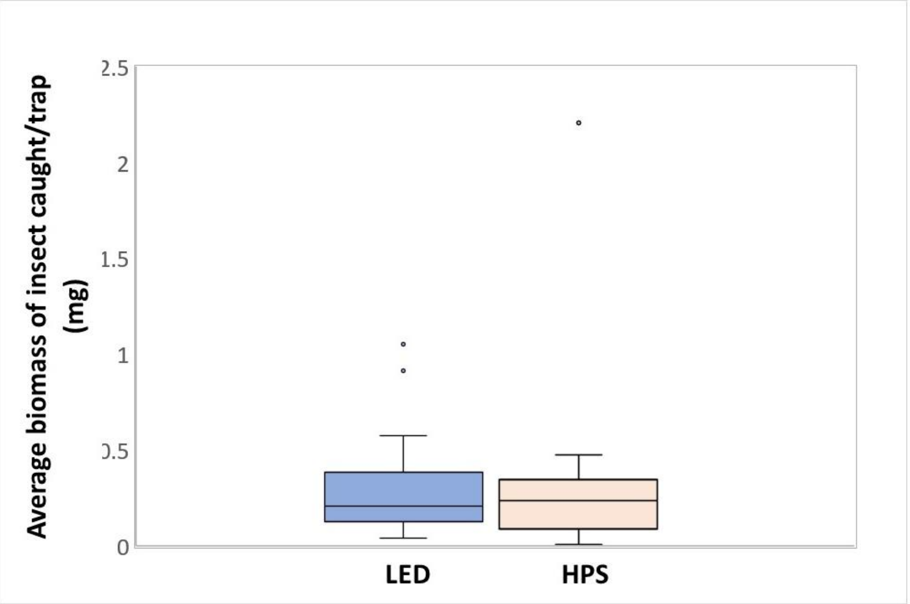

Figure 8: Biomass found at the traps of each light type.

The biomass of the insects found in LED and HPS light types do not differ from each other. ($F_{1.9}$=2.30, P=0.16).

The light type did not affect the estimated biomass ($F_{1.9}$=2.30, P=0.16) (Fig. 8). In fact, none of the variables in my model affected average biomass. However, these results were influenced by three outliers, i.e., three very heavy insects. Repeating the analysis after excluding the outliers mentioned earlier yielded a different conclusion. Although light type still did not influence biomass ($F_{1.9}$=0.48, P=0.50), light intensity had a negative relationship with average biomass of the insects captured (R^2=0.09, Adjusted R^2=0.07, $F_{1,45}$ = 4.35, P= 0.04).

Discussion

Insect richness, Abundance and Light Types

Contrary to my hypothesis where more insects are attracted to white light and thus toward LED lamps, I found higher richness and abundance of insects in HPS light traps. It turns out that the wavelength of the light is more important than colour temperature in determining the attractiveness of the light to the insects. Indeed, studies have attributed the cause of insect behaviour to wavelength-specific rhodopsin in the photoreceptor cells in their eyes[13]. Although the presence of different rhodopsins in insect photoreceptor cells allows them to perceive light over a wide range of wavelengths[49], all insects express rhodopsin that absorbs maximally in the UV range of light[14]. To illustrate this, various species share an attraction to UV light while exhibiting more variation in their reactions to other wavelengths. For example, dipterans are attracted to UV[50], blue and green light[51,52], whereas honeybees (Hymenoptera)[34] and lepidopterans exhibit positive phototaxis to UV[53] and blue light[15]. Hence, the UV emission of lamps generates a stronger attraction to artificial lights in insects as compared to the effects of the lamp's visibility[15]. Coincidentally, HPS lights have peak UV emission while LED light emissions tend more toward blue wavelengths[2]. These findings are consistent with the results of this study, where more insects were found in traps with HPS lights as compared to LED lights. My results also concur with those by Rydell (1992) and Blake et al. (1994), in which more insects were attracted to “white” mercury-vapour lamps, which emit UV radiation, than to LPS lamps, which emit orange light[20,27]. As such, positive phototaxis observed in insects seems to be positively related to the dominance of UV wavelengths in light emission.

Moreover, I captured proportionally more leafhoppers (Cicadellidae) and ants (Formicidae) in LED light traps. Some members of the leafhopper family display attraction to longer light wavelengths[54] due to tail sensitivity of green-absorbing visual pigments, while some ant species, such as *Bombus hypnorum* expressed greater spectral sensitivity in the blue spectrum of visible light[55], which may to help to explain why more leafhoppers and ants are attracted to longer light wavelengths emitted by LED light, as compared to the shorter UV light wavelengths emitted by HPS lights. Furthermore, this also provides more evidence that positive phototaxis in insects is caused by the spectral emission of artificial lights.

Abundance and Rainfall

The positive relationship between rainfall and number of the insects caught per trap could reflect the relationship between seasonality and abundance of insects. Indeed, my study period coincided with the change in seasonality, namely the onset of the monsoon. Marked increases in monthly rainfall are associated with dramatic increases in the abundance of some tropical insects[56], e.g., flies (Diptera) in Columbia[57], beetles (Coleoptera) in Brazil and tree insects in Costa Rica[58]. Of course, it is essential to acknowledge that rainfall does not affect all insects the same way, and seasonality patterns can differ among insect orders[59] and even species[60]. In other words, the monsoon in Singapore might not be favourable for all major groups[61]. For example, although rainfall can act as a seasonal cue for mating for some insects[62], it can also represent a form of water stress for others[63]. Therefore, my results could be biased toward a few taxa that flourish in the wet season. I was not able to ascertain the absolute effect of rainfall on every family's relative abundance as different genera within the same family (e.g. Chironomidae) could show varying responses to rain[64,65].

Biomass of Insect and Light Type

Contrary to my expectations where heavier insects are captured at LED lights, there was no difference in biomass of insects between both light types. Instead, biomass was negatively affected by light intensity, regardless of light type (after removing outliers).

Light intensity exerts a plant-mediated effect on insects[66]. High light intensity could disrupt photosynthesis[67] and induce the production of secondary metabolites, such as plant defensive chemicals[66]. The growth-differentiation balance hypothesis predicts that such resource allocation to defence comes at the expense of growth via lowered production of primary metabolites[68]. The secretion of defensive chemicals such as trichomes in *Lycopersicon* serves to repel insects such as lepidopterans, aphids and leaf miners[69], thus altering their feeding behaviour. Consequentially, insects are less likely to feed in the presence of high light intensity, as increased production of defence chemicals and a reduction of primary metabolites makes the leaf material less palatable for herbivores[70]. The reverse also holds true, where caterpillars have been observed to increase their feeding rates on physically shaded plants such as in tomato and tobacco[66]. In short, increasing light intensity could mean decreased food availability for the insects, and thus affecting their biomass.

As a whole, my results found that LED lights attract fewer insects than HPS lights do. On the contrary, the similar studies conducted in New Zealand[25] and Germany[19] captured more insects than HPS lamps.

The disparity in results could reflect the influence of biome. Plant species richness is, on average, five to ten times higher in tropical than in temperate forests[71], and with a higher diversity of plants comes a greater diversity of insects[71]. Evidence from phylogenetic analysis of ants (Formicidae) has suggested that biodiversification started in Tropics,

making Tropics the “museum and cradle of diversity” [72]. Consequentially, taxa of ants present in the Tropics are older and have a more extensive geographic range in their size[19]. Therefore, due to the difference in evolutionary history, insect in the temperate and tropical zone could have different developmental adaptations could have resulted in different spectral sensitivity to light, which then led to the result deviations observed between this experiment and the previous studies.

Overall levels of light pollution

The ecological impacts of streetlights are well-documented in diverse taxa, including vertebrates and invertebrates, but they should differ according to context, especially, overall light pollution. In other words, it stands to reason that the greater the overall level of ambient light at night, the lower the attractive power of an individual light source. ALAN in Singapore is pervasive, and so on any given night, there is severe sky glow. Indeed, as mentioned, Singapore was named the most light-polluted nation[30]. We also know that ALAN is implicated in behavioural changes in certain arthropods, which have substantially adapted to light pollution. For example, some species of diurnal jumping spiders have shifted to exploiting available light after dark, essentially becoming facultatively nocturnal in urban environments[73]. It is, therefore, reasonable to hypothesise that the insects in Singapore may have adjusted to pervasive light pollution to the point that their behaviours have already deviated significantly from the norm. In crickets, the changes in the environmental light cycle have been known to alter the waveform of the circadian pacemaker. Downstream activation of the light-sensitive neurons by pacemaker change the length of the perceived day, resulting in specific behavioural responses[74]. Furthermore, studies involving flies revealed permanent disruptions to their circadian rhythm in the presence of light[75]. Flies reared in persistent light had a shorter circadian period as compared to flies reared in darkness, substantiating my hypothesis that the behaviour of insects in Singapore may have already deviated from insects native to other countries which might be more dimly-lit.

Implications

There is no question that worldwide LED retrofits are worthwhile endeavours when it comes to mitigating climate change. Most importantly, in this study, an unintentional benefit of the LED retrofit is insect conservation, i.e., a reduction in mortality due to streetlights.

However, there are also implications for predators of phototactic insects that have adapted to using outdoor lights as foraging grounds (e.g., certain species of insectivorous bats). Specifically, the retrofit could reduce the profitability of these hunting grounds. LED lamps attracted fewer insects without a concomitant increase in biomass. This means that for any predators that continue to hunt at these lights the search and handling time per prey item are likely to increase, and energy intake per successful attack is expected to decrease.

Moreover, if they are less attractive to insects, as I found, then there are implications for human-insect interactions. For example, Leishmaniasis is one of the world's most important tropical vector-borne diseases, and the vectors are sand flies. Sand flies are frequently attracted to artificial lights. Thus, outdoor lights may promote interactions between humans and pathogen vectors, and hence, increase transmission[22]. If LED lighting really does attract fewer insects, then the retrofit could contribute to reducing the likelihood of disease transmission.

Future work

The most prominent shortcoming of this study is that LTA would not allow me to mount insect traps on 6m lamp posts, i.e., streetlight height. Instead, I was restricted to sites with 3.5m lamp posts along footpaths. The problem is the well-documented vertical stratification of insect assemblages[76]. Indeed, during my 2-hour sampling periods, I did occasionally see moths flying around the taller streetlights that were near my sites, but I never recorded them at the 3.5m lamp posts. I suspect that insect assemblages around the taller street lights differs to those around the shorter lamps I sampled, and it could be that the relative attractiveness of the two light types differs between both heights of lights. Therefore, I strongly suggest that the ecological impact of the LED retrofit be reassessed using the same study design, but at these 6m street lights.

It is also possible that the more considerable heat emitted by HPS lights than by LED lights can act as a thermal attractant for some dipterans[77]. I did not measure thermal emissions, but I suspect that thermal contrast between a warm light and the surrounding environment varies with ambient temperature, and would be less pronounced in a tropical environment, as opposed to in a temperate climate.

Besides, I did not identify species or measure the height of surrounding vegetation, which could have been very influential on the insect assemblage in each site. The presence of host plants may influence the reproductive[78] and foraging strategies[79] of insects, thereby affecting the diversity, richness, abundance and biomass of the insects captured.

Therefore, I plan to survey of surrounding vegetation this summer.

Finally, this is one study in one tropical city – an anomalous one for its level of urbanisation and light pollution. The results should therefore not be generalised to represent all LED retrofits in the Tropics. Instead, I recommend similar studies in the Old and New World Tropics and locales with different levels of light pollution.

Conclusion

This study highlights the effects of LED retrofit on the insect population in Singapore. Considering the diverse ecological roles that insects play, the profound adverse effects of artificial lights on insects could result in a cascading impact on the entire ecosystem. While life in modern society seems inconceivable without artificial lights, light pollution is frequently caused due to poor design or overuse. Therefore, LED lighting presents itself as an attractive and environmentally friendly alternative. While my study addresses some of the ecological effects of LED about the insect population, its impact on higher trophic levels remains unknown.

References

1. Longcore, T. & Rich, C. Ecological light pollution. *Frontiers in Ecology and the Environment* **2,** 191–198 (2004).
2. Longcore, T. *et al.* Tuning the white light spectrum of light emitting diode lamps to reduce attraction of nocturnal arthropods. *Philos. Trans. R. Soc. B Biol. Sci.* **370,** 20140125–20140125 (2015).
3. Hölker, F. *et al.* The dark side of light : a transdisciplinary research agenda for light. *Ecol. Soc.* **15,** 13 (2010).
4. Stone, E. L., Jones, G. & Harris, S. Conserving energy at a cost to biodiversity? Impacts of LED lighting on bats. *Glob. Chang. Biol.* **18,** 2458–2465 (2012).
5. Cho, S., Chang, S. & Jo, I. The solid-state drive technology, today and tomorrow. in *Proceedings - International Conference on Data Engineering* **2015–May,** 1520–1522 (2015).
6. Gereffi, G., Lowe, M., Ayee, G., Frederick, S. & Gui, L. LED Lighting. *Ieee Spectr.* **48,** 1–15 (2011).
7. Li, F., Chen, Y., Liu, Y. & Chen, D. Comparative in situ study of LEDs and HPS in road lighting. *LEUKOS - J. Illum. Eng. Soc. North Am.* **8,** 205–214 (2012).
8. Dunn, R. R. Modern insect extinctions, the neglected majority. *Conserv. Biol.* **19**, 1030–1036 (2005).
9. Samways, M. J. Insects in biodiversity conservation: some perspectives and directives. *Biodivers. Conserv.* **2,** 258–282 (1993).
10. Chung Kim, K. Biodiversity, conservation and inventory: why insects matter. *Biodivers. Conserv.* **2,** 191–214 (1993).
11. Campobasso, C. P., Vella, G. D. & Introna, F. Factors affecting decomposition and Diptera colonization. *Forensic Sci. Int.* **120,** 18–27 (2001)
12. Robinson, G. E. Regulation of division of labor in insect societies. *Annu. Rev. Entomol.* **37,** 637–665 (1992).
13. Poiani, S., Dietrich, C., Barroso, A. & Costa-Leonardo, A. M. Effects of residential energy-saving lamps on the attraction of nocturnal insects. *Light. Res. Technol.* **47,** 338–348 (2015).
14. Hamdorf, K., Schwemer, J. & Gogala, M. Insect visual pigment sensitive to ultraviolet light. *Nature* **231,** 458–459 (1971).
15. Barghini, A. & De Medeiros, B. A. S. UV radiation as an attractor for insects. *LEUKOS - J. Illum. Eng. Soc. North Am.* **9,** 47–56 (2012).
16. Scherer, C. & Kolb, G. The influence of color stimuli on visually controlled behavior in Aglais urticae L. and Pararge aegeria L. (Lepidoptera). *J. Comp. Physiol. A* **161,** 891–898 (1987).
17. Verheijen, F. J. The mechanisms of the trapping effect of artificial light sources upon animals. *Arch. Néerlandaises Zool.* **13,** 1–107 (1960).

18. Eisenbeis, G. & Hänel, A. Light pollution and the impact of artificial night lighting on insects. In *Ecology of cities and towns: A comparative approach* (eds McDonnell, M. J., Hahs, A. K. & Breuste, J.). 243–263 (Cambridge Univ. Press, 2009).

19. Eisenbeis, G. Artificial night lighting and insects: attraction of insects to streetlamps in a rural seting in Germany. In *Ecology consequences of artificial night lighting* (eds Longcore, T. & Rich C.). 281–304 (Island Press,2006).

20. Rydell, J. Exploitation of insects around streetlamps by bats in Sweden. *Funct. Ecol.* **6,** 744 (1992).

21. Hori, M., Shibuya, K., Sato, M. & Saito, Y. Lethal effects of short-wavelength visible light on insects. *Sci. Rep.* **4,** (2014).

22. Barghini, A. & de Medeiros, B. A. S. Artificial lighting as a vector attractant and cause of disease diffusion. *Environ. Health Perspect.* **118,** 1503–1506 (2010).

23. Steele, R. Strategically speaking: LCD backlights and lighting drive largest growth yet seen in HB-LED market - LEDs. *LED magazine* **7,** 3–26 (2010).

24. Peters, L. Lighting market report predicts strong growth for LED lighting. *LED Magazine* **8,** 1–32 (2011).

25. Pawson, S. M. & Bader, M. K.-F. LED lighting increases the ecological impact of light pollution irrespective of color temperature. *Ecol. Appl.* **24,** 1561–1568 (2014).

26. Eisenbeis, G. & Eick, K. Studie zur anziehung nachtaktiver insekten an die Straßenbeleuchtung unter einbeziehung von LEDs. *Natur. und Landschaft.* **86,** 298–306 (2011).

27. Blake, D., Hutson, A. M., Racey, P. A., Rydell, J. & Speakman, J. R. Use of lamplit roads by foraging bats in southern England. *J. Zool.* **234,** 453–462 (1994).

28. Sarma, S. S. S., Nandini, S. & Gulati, R. D. Life history strategies of cladocerans: Comparisons of tropical and temperate taxa. *Hydrobiologia* **542,** 315–333 (2005).

29. Feder, M. E. Environmental variability and thermal acclimation in Neotropical and temperate zone species. *Physiol. Zool.* **51,** 7–16 (1978).

30. Falchi, F. *et al.* The new world atlas of artificial night sky brightness. *Sci. Adv.* **2,** 1-26 (2016).

31. Corlett, R. T. The ecological transformation of Singapore, 1819-1990. *J. Biogeogr.* **19,** 411–420 (1992)..

32. Abdullak, Z. LTA installing smarter, energy-saving street lights. *The Straits Times* http://www.straitstimes.com/singapore/lta-installing-smarter-energy-saving-street-lights (4 January 2017).

33. Rutowski, R. L., Gislén, L. & Warrant, E. J. Visual acuity and sensitivity increase allometrically with body size in butterflies. *Arthropod Struct. Dev.* **38,** 91–100 (2009).

34. Menzel, R. & Greggers, U. Natural phototaxis and its relationship to colour vision in honeybees. *J. Comp. Physiol. A* **157,** 311–321 (1985).

35. Triplehorn, C. & Johnson, N. *Borror and Delong's Introduction of the Study of Insects*. 7th edn. (Brooks Cole, 2004).

36. Marshall, S. A. *Flies: The Nature History and Diversity of Diptera*. (Firefly Books, 2012).

37. Goulet, H. & Huber, J. T. *Hymenoptera of the World: An identification guide to families*. (Research Branch, Agricultural Canada Publication. Canada Communication Group-Publishing, 1993).

38. CSIRO. *The Insects of Australia: a textbook for students and research workers*. (Cornell University Press, 1991).

39. Sample, B. E., Cooper, R. J., Greer, R. D. & Whitmore, R. C. Estimation of insect biomass by length and width. *Am. Midl. Nat.* **129,** 234–240 (1993).

40. Hodar, J. A. The use of regression equations for estimation of arthropod biomass in ecological studies. *Acta Oecologica-International J. Ecol.* **17,** 421–433 (1996).

41. Gruner, D. Regressions of length and width to predict arthropod biomass in the Hawaiian Islands. *Pacific Sci.* **57,** 325–336 (2003).

42. Benke, A. C., Huryn, A. D., Smock, L. A. & Wallace, J. B. Length-Mass Relationships for Freshwater Macroinvertebrates in North America with Particular Reference to the Southeastern United States. *J. North Am. Benthol. Soc.* **18,** 308–343 (1999).

43. Sabo, J. L., Bastow, J. L. & Power, M. E. Length-Mass relationships for adult aquatic and terrestrial invertebrates in a California watershed. *J. North Am. Benthol. Soc.* **21,** 336 (2002).

44. NEA. Historical Daily Records. http://www.nea.gov.sg/weather-climate/climate/historical-daily-records. (accessed, 30 March 2018).

45. Inc., G. Google Maps 9.73.3. (2018).

46. Delettre, Y. R. & Morvan, N. Dispersal of adult aquatic Chironomidae (Diptera) in agricultural landscapes. *Freshw. Biol.* **44,** 399–411 (2000).

47. Fattorini, S. Insect extinction by urbanization: A long term study in Rome. *Biol. Conserv.* **144,** 370–375 (2011).

48. SAS Institute Inc., Cary, N. SAS. *SAS Institute Inc., Cary, NC* 8640 (2018).

49. Daly, H. V., Doyen, J. T. & Purcell, A. H. *Introduction to insect biology and diversity*. (Oxford University Press, 1998).

50. Burkett, D. A., Butler, J. F. & Kline, D. L. Field evaluation of colored light-emitting diodes as attractants for woodland mosquitoes and other diptera in north central Florida. *Joumal Am. Mosq. Control Assoc.* **14,** 186–195 (1998).

51. Burkett, D. A. & Butler, J. F. Laboratory evaluation of colored light as an attractant for female *Aedes aegypti, Aedes albopictus, Anopheles quadrimaculatus,* and *Culex nigripalpus*. *Florida Entomol.* **88,** 383–389 (2005).

52. Bishop, A. L., Worrall, R., Spohr, L. J., McKenzie, H. J. & Barchia, I. M. Response of Culicoides spp. (Diptera: Ceratopogonidae) to light-emitting diodes. *Aust. J. Entomol.* **43,** 184–188 (2004).

53. Plummer, K. E., Hale, J. D., O'Callaghan, M. J., Sadler, J. P. & Siriwardena, G. M. Investigating the impact of street lighting changes on garden moth communities. *J. Urban Ecol.* **2,** 1–10 (2016).

54. Wakakuwa, M., Stewart, F., Matsumoto, Y., Matsunaga, S. & Arikawa, K. Physiological basis of phototaxis to near-infrared light in *Nephotettix cincticeps*. *J Comp Physiol A* **200,** 527–536 (2014).

55. Peitsch, D. *et al.* The spectral input systems of hymenopteran insects and their receptor-based colour vision. *J. Comp. Physiol. A* **170,** 23–40 (1992).

56. Kato, M. *et al.* Seasonality and vertical structure of light-attracted insect communities in a dipterocarp forest in Sarawak. *Res. Popul. Ecol. (Kyoto).* **37,** 59–79 (1995).

57. Tanaka, L. K. & Tanaka, S. K. rainfall and seasonal changes in arthropod abundance on a tropical oceanic island. *Biotropica* **14,** 114–123 (1982).

58. Buskirk, R. E. & Buskirk, W. H. Changes in arthropod abundance in a Highland Costa Rican forest. *Am. Midl. Nat.* **95,** 288–298 (1976).

59. Anu, A., Sabu, T. K. & Vineesh, P. Seasonality of litter insects and relationship with rainfall in a wet evergreen forest in south western ghats. *J. Insect Sci.* **9,** 1–10 (2009).

60. Wolda, H. Fluctuations in abundance of tropical insects. *Am. Nat.* **112,** 1017–1045 (1978).

61. Denlinger, D. L. Seasonal and annual variation of insect abundance in the Nairobi National Park, Kenya. *Biotropica* **12,** 100-106 (1980).

62. van Huis, A. Cultural significance of termites in sub-Saharan Africa. *J. Ethnobiol. Ethnomed.* **13,** (2017).

63. Schowalter, T. D., Lightfoot, D. C. & Whitford, W. G. Diversity of arthropod responses to host-plant water stress in a desert ecosystem in southern new mexico. *Am. Midl. Nat.* **142,** 281–290 (1999).

64. Ocampo Righi-Cavallaro, K., Roche, K. F., Froehlich, O. & Cavallaro, M. R. Structure of macroinvertebrate communities in riffles of a Neotropical karst stream in the wet and dry seasons. *Acta Limnol. Bras. Biol. Limnol.* **22,** 306–316 (2010).

65. Angradi, T. R. Fine sediment and macroinvertebrate assemblages in appalachian streams: A field experiment with biomonitoring applications. *J. North Am. Benthol. Soc.* **18,** 49–66 (1999).

66. Vänninen, I., Pinto, D. M., Nissinen, A. I., Johansen, N. S. & Shipp, L. In the light of new greenhouse technologies: Plant-mediated effects of artificial lighting on arthropods and tritrophic interactions. *Annals of Applied Biology* **157,** 393–414 (2010).

67. Lambers, H., Chapin, F. S. & Pons, T. L. *Plant Physiological Ecology*. 2nd edn (Springer, 2008).

68. Herms, D. A. & Mattson, W. J. The dilemma of plants: to grow or defend. *Q. Rev. Biol.* **67,** 283–335 (1992).

69. Nihoul, P. Do light intensity, temperature and photoperiod affect the entrapment of mites on glandular hairs of cultivated tomatoes? *Exp. Appl. Acarol.* **17,** 709–718 (1993).

70. Roberts, M. R. & Paul, N. D. Seduced by the dark side: integrating molecular and ecological perspectives on the influence of light on plant defence against pests and pathogens. *New Phytol* **170,** 677–699 (2006).

71. Novotny, V. *et al.* Why are there so many species of herbivorous insects in tropical rainforests? *Science.* **313,** 1115–1118 (2006).

72. Moreau, C. S. & Bell, C. D. Testing the museum versus cradle tropical biological diversity hypothesis: phylogeny, diversification, and ancestral biogeographic range evolution of the ants. *Evolution (N. Y).* **67,** 2240–2257 (2013).

73. Gaston, K. J., Bennie, J., Davies, T. W. & Hopkins, J. The ecological impacts of nighttime light pollution: A mechanistic appraisal. *Biol. Rev.* **88,** 912–927 (2013).

74. Tomioka, K. & Chiba, Y. Light cycle during post-embryonic development affects adult circadian parameters of the cricket *(Gryllus bimaculatus)* optic lobe pacemaker. *J. Insect Physiol.* **35,** 273–276 (1989).

75. Tomioka, K., Uwozumi, K. & Matsumoto, N. Light cycles given during development affect freerunning period of circadian locomotor rhythm of period mutants in *Drosophila melanogaster*. *J. Insect Physiol.* **43,** 297–305 (1997).

76. Johnson, C. G. The distribution of insects in the air and the empirical relation of density to height. *Source J. Anim. Ecol.* **26,** 479–494 (1957).

77. Wakefield, A., Broyles, M., Stone, E. L., Jones, G. & Harris, S. Experimentally comparing the attractiveness of domestic lights to insects: Do LEDs attract fewer insects than conventional light types? *Ecol. Evol.* **6,** 8028–8036 (2016).

78. Awmack, C. S. & Leather, S. R. Host plant quality and fecundity in herbivorous insects. *Annu. Rev. Entomol.* **47,** 817–844 (2002).

79. Scheirs, J. Integrating optimal foraging and optimal oviposition theory in plant-insect research. *Oikos* **96,** 187–191 (2002).
